# Metagenomics analysis of the neonatal intestinal resistome

**DOI:** 10.3389/fped.2023.1169651

**Published:** 2023-06-16

**Authors:** Stefano Leo, Omer F. Cetiner, Laure F. Pittet, Nicole L. Messina, William Jakob, Laurent Falquet, Nigel Curtis, Petra Zimmermann

**Affiliations:** ^1^ Department for Community Health, Faculty of Science and Medicine, University of Fribourg, Fribourg, Switzerland; ^2^ Department of Paediatrics, Fribourg Hospital, Fribourg, Switzerland; ^3^ Istanbul Faculty of Medicine, Istanbul University, Istanbul, Türkiye; ^4^ Department of Paediatrics, The University of Melbourne, Parkville, VIC, Australia; ^5^ Infectious Diseases Research Group, Murdoch Children’s Research Institute, Parkville, VIC, Australia; ^6^ Pediatric Infectious Diseases Unit, Geneva University Hospitals and Faculty of Medicine, Geneva, Switzerland; ^7^ Microbiology Laboratory, Fribourg Hospital, Fribourg, Switzerland; ^8^ Department of Biology, University of Fribourg and Swiss Institute of Bioinformatics, Fribourg, Switzerland; ^9^ Infectious Diseases Unit, The Royal Children’s Hospital Melbourne, Parkville, VIC, Australia

**Keywords:** antibiotic resistance, mobilome, stool, microbiome, microbiota, gut, infant

## Abstract

**Introduction:**

The intestinal microbiome forms a major reservoir for antibiotic resistance genes (ARGs). Little is known about the neonatal intestinal resistome.

**Objective:**

The objective of this study was to investigate the intestinal resistome and factors that influence the abundance of ARGs in a large cohort of neonates.

**Methods:**

Shotgun metagenomics was used to analyse the resistome in stool samples collected at 1 week of age from 390 healthy, term-born neonates who did not receive antibiotics.

**Results:**

Overall, 913 ARGs belonging to 27 classes were identified. The most abundant ARGs were those conferring resistance to tetracyclines, quaternary ammonium compounds, and macrolide-lincosamide-streptogramin-B. Phylogenetic composition was strongly associated with the resistome composition. Other factors that were associated with the abundance of ARGs were delivery mode, gestational age, birth weight, feeding method, and antibiotics in the last trimester of pregnancy. Sex, ethnicity, probiotic use during pregnancy, and intrapartum antibiotics had little effect on the abundance of ARGs.

**Conclusion:**

Even in the absence of direct antibiotic exposure, the neonatal intestine harbours a high abundance and a variety of ARGs.

## Introduction

The intestinal microbiome is a complex ecosystem with a large and diverse community of microbes. This forms a major reservoir for antibiotic resistance genes (ARGs), which is also called the intestinal resistome (1–3). Neonates are rapidly colonised after birth, acquiring their intestinal microbiome from their mothers and other contacts, through milk and from the environment. The most abundant bacterial families in the neonatal intestine are Enterobacteriaceae, Bifidobacteriaceae, Bacteroidaceae, Staphylococcaceae, and Clostridiaceae (4). These bacteria are a major reservoir of clinically relevant ARGs (5–7). Antibiotics influence the composition of intestinal microbiome and resistome (8) but ARGs can also be detected in the absence of antibiotic treatment (9, 10).

Neonates are highly vulnerable to infections, which are often associated with significant morbidity and mortality. Antibiotic-resistant, especially multidrug-resistant, bacteria pose a major threat to neonatal health. It is estimated that, annually, over 200,000 neonatal deaths occur due to infections with antibiotic-resistant pathogens (11). Moreover, infections with antibiotic-resistant pathogens are also associated with higher rates of complications, prolonged hospital stay, increased cost, and worse outcomes. Little is known about the neonatal intestinal resistome.

In this study, we used shotgun metagenomics to investigate the intestinal resistome and the factors that influence the abundance of ARGs in a large cohort of neonates.

## Methods

### Study design and participants

A subset of neonates from The Melbourne Infant Study: BCG for Allergy and Infection Reduction (MIS BAIR) who did not receive antibiotics during the first week of life were included (12). In the MIS BAIR trial, 1,272 healthy neonates were randomised to Bacille Calmette–Guérin (BCG) immunisation or not to investigate whether the vaccine protects against childhood infection, allergy, and asthma. Inclusion criteria were: gestational age greater than 32 weeks, birth weight greater than 1,500 g, absence of symptoms or signs of illness, and no contraindication to BCG. Data on demographics, pregnancy, and the perinatal period were collected through questionnaires.

### Stool collection

On each of the first 10 days of life, stool samples from the first bowel movement of the day were collected into empty sterile tubes by parents (with date and time recorded), who were given detailed oral and written instructions about the collection technique. Samples were stored in domestic freezers (−18°C or below). They were collected from parents’ home by a study team member and kept frozen during transportation to the laboratory, where they were aliquoted for storage at −80**°**C. One stool sample collected between day 5 and 7 of life was analysed for each neonate.

### DNA extraction and metagenomic shotgun sequencing

Aliquots of stools were thawed at room temperature and 100 mg of stool was used for DNA extraction. The FastDNA™ SPIN Kit for Soil (MP Biomedicals, Illkirch-Graffenstaden, France) was used according to manufacturer's instructions. DNA concentrations were measured using Qubit™ dsDNA High Sensitivity Assay kits (Life technologies, California, United States). The mean DNA concentration in samples was 367 ng; the DNA in the negative control was below the detection limit (<0.01 ng/µl) and was therefore not sequenced. Sequencing libraries with a final insert size of approximately 600 bp were prepared with Nextera DNA Flex library preparation kits (Illumina, San Francisco, United States). Illumina PhiX DNA was added to the libraries. Paired sequencing (2 × 149 bp) was done on a NextSeq 550 system (Illumina) with high-output flow cells. Bacterial and fungal even-distributed mock communities (Gut Microbiome Whole cell Mix MSA-2006™ and Mycobiome Whole Cell Mix, respectively, ATCC, Manassas, United States) were sequenced as positive controls.

### Bioinformatic analyses

#### Quality filtering and removal of human-mapping reads

Reads were checked for quality with FastQC v0.11.7 (http://www.bioinformatics.babraham.ac.uk/projects/fastqc/). Filtering of low-quality reads and trimming were done with Trimmomatic v0.39 (13) with the following settings: SLIDINGWINDOW:20:28, MINLEN:100. Sequences mapping to the human genome (vGRCh38.p11) with a confidence score of more than 0.1 were removed with Kraken v2.0.9-beta (14).

#### Identification of bacteria and bacteriophages

Quality-filtered non-human reads were analysed with Kraken2 and mapped to bacterial and bacteriophagic genomes with confidence score higher than 0.1. Read taxonomic assignment was re-estimated with Bracken v2.6.2 (14, 15) at phylum, family, genus, and species levels.

To calculate the relative abundance of a bacterial taxon in a given sample, the number of reads assigned to the taxon was divided by the total number of reads classified at the same taxonomic level in the sample. The relative abundance of bacteriophages belonging to the *Caudovirales* order, the most represented taxon in the human intestine (16, 17), was expressed in counts per million (CPM). CPMs were obtained by normalising reads mapping to bacteriophagic genomes with the total number of quality-filtered non-human reads.

#### Detection of antibiotic resistance genes

ResFinder was used to detect ARGs as the database contains all the major clinically relevant ARGs. Quality-filtered non-human forward (R1) and reverse (R2) FASTQ files were mapped to the ResFinder gene database v4 (18) with bowtie2 v2.3.1 (19) with the following settings: -D 20 -R 3 -N 1 -L 20 -i S,1,0.50 (20). Alignment SAM files were sorted and indexed with SAMtools v1.10 (21). Read counts for a given ResFinder sequence in each sample were obtained with bedtools multicov (BEDTools v2.29.2) (22) with the following settings: -p -q 1.

Read counts were normalised to the total number of reads assigned by Kraken2/Bracken to bacterial species and the length of each ARG sequence (expressed in kb). The ratio was then multiplied by one million. Normalised counts for genes were obtained by adding normalised counts of all the ResFinder sequences of a given ARG. The matching of each ResFinder sequence to a given antibiotic class was obtained from the “phenotype.txt” file contained in the ResFinder database. ARG normalised counts were used for further analyses.

### Statistical analysis

Statistics were performed in the R software version 4.2.1. The codes can be found in the Supplementary File 1. The correlation between ARGs and bacterial species composition detected by Kraken2/Bracken was analysed with the Mantel test from the R package vegan v2.6-2 by using 999 permutations. ARG normalised counts and the relative abundance of species were first analysed with the *vegdist* function by choosing the Horn–Morisita index and obtained distance matrixes were then submitted to the Mantel test.

The bacterial (genus and species) and resistome profiles were analysed by principal coordinate analyses (PCoAs). The relative abundance was square-root-transformed, and a distance matrix was computed based on the Bray–Curtis dissimilarity index with the *vegdist* function (vegan). The matrix was analysed with the *betadisper* function (vegan).

The association between different factors and the bacterial (phylum, family, genus, and species) or resistome composition was investigated with permutational multivariate analysis of variance (PERMANOVA) test implemented in the *adonis2* function (vegan) by using Bray–Curtis distances and 9,999 permutations.

Ecological indices (richness, Shannon, and Simpson diversity) were computed with the *diversity* function (vegan). Rarefaction to 35,000 reads was performed for species composition with the *rrarefy* function (vegan). Differentially abundant taxa were identified by the Wilcoxon rank sum test, and differentially abundant ARGs with DESeq2 v1.36.0 (23). To assess differential abundance of taxa, the median values of relative abundance were used to calculate fold-change, which was then transformed to the log_2_ scale. This approach enabled comparisons of the abundance of each taxon across multiple samples, while minimising the impact of outliers.

For ARGs, log_2_-transformed fold changes were provided by DESeq2. Ecological indices were performed on rounded normalised counts of ARGs. DESeq2 was run on pseudo-counts obtained by adding 1 to the rounded normalised counts.

### Reference databases

The ResFinder database version 4 consists of 3,139 unique sequences. When adding up the sequences for the same gene (or variants, as for example for *bla_OXA_*, *bla_TEM_*, etc.), this corresponded to 2,534 ARGs. For Kraken2 analyses, the human genome (vGRCh38.p11), 15,009 bacterial, and 11,833 viral genomes were used. All reference sequences were downloaded from NCBI RefSeq on 3 March 2022.

### Ethics

Written informed consent was obtained from parents or guardians of all neonates. The study was approved by the Royal Children's Hospital Human Research Ethics Committee [HREC, authorisation (53543)]. MIS BAIR is registered with the Australian New Zealand Clinical Trials Registry (1051228) and the U.S. National Institutes of Health (NCT01906853).

## Results

In total, 390 healthy, term-born neonates who did not receive antibiotics during the first week of life were included. The median gestational age at birth was 39.3 [interquartile range (IQR) 38.4–40.3] weeks and the median birth weight was 3,440 (IQR: 3,122–3,748) grams.

Of these neonates, 33% (129/390) were born by Caesarean section (CS) and 50% (195/390) were female. During the first week of life, 33% (128/390) neonates received formula milk and one neonate received probiotics.

Of the mothers, 18% (71/390) took probiotics during pregnancy and 12% (48/390) received at least one course of antibiotics during the last trimester of pregnancy. Amoxicillin was the most frequently used antibiotic (50%, 30/60), followed by cephalexin (13%, 8/60) and penicillin (7%, 4/60). In total, 23% (90/390) mothers received intrapartum antibiotics. The most frequently used drug was penicillin (52%, 47/90), followed by amoxicillin (33%, 30/90). The main two reasons for intrapartum antibiotic use were anogenital group B *Streptococcus* carriage (GBS) (58%, 52/90) and prolonged rupture of membranes (34%, 31/90) (Table 1).

**Table 1:** Cohort characteristics.

Factors	*n* (%) or median (IQR) *n* = 390
Caesarean section	129 (33)
Gestation, weeks	39.3 (38.4–40.3)
Birth weight, g	3,440 (3,122–3,748)
Sex, female	195 (50)
Caucasian (3 or 4 grandparents)	307 (79)
Asian (3 or 4 grandparents)	17 (4)
Mixed Caucasian and Asian	19 (5)
Other	47 (12)
Formula milk before 7 days of life	128 (33)
Probiotics during first 7 days of life	1 (0.3)
Probiotics during pregnancy (any trimester)	71 (18)
Antibiotics during pregnancy (last trimester)	48 (12)
No. of antibiotic courses	
1	40/48 (83)
2	6/48 (13)
3	1/48 (2)
5	1/48 (2)
Amoxicillin	30/60 (50)
Amoxicillin/clavulanic acid	1/60 (2)
Azithromycin	1/60 (2)
Ceftriaxone	1/60 (2)
Cephalexin	8/60 (13)
Cephazolin	2/60 (3)
Ciprofloxacin	1/60 (2)
Clindamycin	1/60 (2)
Erythromycin	2/60 (3)
Flucloxacillin	2/60 (3)
Metronidazole	1/60 (2)
Penicillin	4/60 (7)
Piperacillin/tazobactam	1/60 (2)
Roxithromycin	1/60 (2)
Unknown	4/60 (7)
Intrapartum antibiotics	90 (23)
Maternal group B Streptococcus carriage	52/90 (58)
Prolonged rupture of membranes	31/90 (34)
Elevated maternal temperature	1/90 (1)
Other	6/90 (7)
Penicillin	47/90 (52)
Amoxicillin/ampicillin	30/90 (33)
Cefotaxime	1/90 (1)
Cephazolin	1/90 (1)
Cephazolin plus metronidazole	1/90 (1)
Clindamycin	9/90 (10)
Unknown	1/90 (1)

IQR, interquartile range.

Antibiotics given as prophylaxis for Caesarean section not included, as given after clamping of the cord.

### Intestinal microbiome composition, richness, and diversity

Delivery mode, gestational age at birth, use of formula milk, and intrapartum antibiotics had a strong effect on the bacterial composition of the intestinal microbiome at all taxonomic levels (PERMANOVA test, *p*-values <0.05; Table 2 and PCoA plots; Figures S1, S2, Supplementary File S2). Birth weight affected the composition at the family, genus, and species levels (*p*-values 0.0075, 0.0022, and 0.0012). Probiotic use during pregnancy affected the composition at the phylum level (*p*-value 0.0413). No association were found between sex, ethnicity, antibiotic use during the last trimester of pregnancy, and the intestinal microbiome composition.

**Table 2:** PERMANOVA tests for bacterial microbiome and resistome composition.

Taxonomy/resistome	Phylum				Family				Genus				Species				Resistome			
Variable	No. of phyla	Pseudo-F^1^	*p*-value	*R* ^2^	No. of families	Pseudo-F^1^	*p*-value	*R* ^2^	No. of genera	Pseudo-F^1^	*p*-value	*R* ^2^	No. of species	Pseudo-F^1^	*p*-value	*R* ^2^	No. of ARGs	Pseudo-F^1^	*p*-value	*R* ^2^
Delivery mode	19	88.99	0.0001	0.1866	212	54.23	0.0001	0.1226	881	51.90	0.0001	0.1180	2,803	41.38	0.0001	0.0964	913	22.79	0.0001	0.0555
Gestational age	19	6.82	0.0013	0.0173	212	7.94	0.0001	0.0201	881	8.46	0.0001	0.0213	2,803	6.87	0.0001	0.0174	913	4.95	0.0001	0.0126
Birth weight	19	1.11	0.347	0.0029	212	3.26	0.0075	0.0083	881	3.61	0.0022	0.0092	2,803	3.37	0.0012	0.0086	913	2.76	0.0017	0.0071
Sex	19	0.41	0.7033	0.0011	212	0.45	0.848	0.0012	881	0.70	0.6947	0.0018	2,803	0.72	0.7429	0.0019	913	0.88	0.5664	0.0023
Ethnicity	19	1.04	0.4128	0.0080	212	0.94	0.5106	0.0072	881	1.22	0.2009	0.0094	2,803	1.21	0.1726	0.0093	913	1.31	0.0807	0.0100
Formula milk during first 7 days of life	19	8.85	0.0001	0.0223	212	8.22	0.0001	0.0207	881	6.76	0.0001	0.0171	2,803	5.20	0.0001	0.0132	913	3.67	0.0001	0.0094
Probiotics during pregnancy (any trimester)	19	2.96	0.0413	0.0076	212	1.73	0.1118	0.0044	881	1.58	0.1183	0.0041	2,803	1.27	0.1969	0.0033	913	1.08	0.3288	0.0028
Antibiotics during pregnancy (last trimester)	19	2.01	0.1303	0.0051	212	1.26	0.2601	0.0032	881	1.15	0.3013	0.0030	2,803	1.10	0.3138	0.0028	913	1.05	0.3611	0.0027
Intrapartum antibiotics	19	6.43	0.0013	0.0164	212	4.08	0.002	0.0105	881	3.68	0.0013	0.0094	2,803	2.75	0.0038	0.0071	913	1.50	0.0863	0.0039

PERMANOVA, permutational multivariate analysis of variance; ARGs, antibiotic resistance genes.

Pseudo-F statistics are used in PERMANOVA to compare within-group to between-group variance (24).

Compared with neonates born by CS, the microbiome of neonates born vaginally had a higher bacterial species richness and diversity (*p*-values <0.001, 0.006, and 0.015 for richness, Shannon, and Simpson diversity, respectively; Figure S3, Supplementary File S2). Species richness and diversity were also higher in neonates of mothers who did not receive intrapartum antibiotics (*p*-values 0.002, <0.001, and <0.001 for richness, Shannon, and Simpson diversity, respectively, Figure S3, Supplementary File S2).

There was no difference in the abundance of bacteriophages between neonates born by CS and those born vaginally (median counts per million 1,300, IQR: 407–3,819, vs. 1,890, IQR: 357–5,350, *p*-value 0.2; Figure S4, Supplementary File S2). A higher abundance of bacteriophages was found in neonates of mothers who received antibiotics during the last trimester of pregnancy (median counts per million 3,021, IQR: 1,073–5,714, vs. 1,444, IQR: 323–4,137, *p*-value 0.01; Figure S4, Supplementary File S2).

Delivery mode, use of formula milk, probiotic use during pregnancy, antibiotic use during the last trimester of pregnancy, and intrapartum antibiotics were associated with at least 1.5-fold difference in the relative abundance of 47 taxa (4 phyla, 9 families, 13 genera, and 21 species) (*p*-values <0.05) (Figures S5–S8, Supplementary File S2). The relative abundance of Firmicutes, Bacteroidetes, and Actinobacteria varied according to delivery mode (*p*-values <0.001; Figure S5, Supplementary File S2). Five of the 390 neonates (one vaginal- and four CS-born) had no reads mapping to Bacteroidetes. In the remaining 385 neonates, the Firmicutes-to-Bacteroidetes ratio was higher in neonates born by CS compared with neonates born vaginally (median 3,324.9, IQR: 1,184.3–14,109.8, vs. 0.6, IQR: 0.1–109.6, *p*-value <0.001). The relative abundance of Actinobacteria was lower in neonates born by CS (median 0.2, IQR: 0.6–1.7, vs. 4.0, IQR: 0.1–28.1; *p*-value <0.001, Figure S5, Supplementary File S2). Neonates born by CS had a higher relative abundance of the families Pasteurellaceae, Veillonellaceae, Streptococcaceae, Staphylococcaceae, and Enterococcaceae (*p*-values <0.01, Figure S6, Supplementary File S2), and the genera *Haemophilus, Veillonella, Streptococcus, Staphylococcus,* and *Enterococcus* (*p*-values <0.01, Figure S7, Supplementary File S2); neonates born vaginally had a higher abundance of Enterobacteriaceae, Tannerellaceae, Bacteroidaceae, and Bifidobacteriaceae (*p*-values <0.01, Figure S6, Supplementary File S2) and the genera *Shigella, Escherichia, Citrobacter, Parabacteroides, Phocaeicola, Bacteroides*, and *Bifidobacterium* (*p*-values <0.0001, Figure S7, Supplementary File S2). Differently abundant species between neonates born by CS and vaginally are detailed in Figure S8, Supplementary File S2.

Neonates who received formula milk during the first week of life had a higher abundance of Enterobacteriaceae, Veillonellaceae, Streptococcaceae, and Enterococcaceae and a lower abundance of Staphylococcaceae and Bacteroidaceae (*p*-values 0.001, 0.022, 0.095, 0.017, <0.001, and <0.001, respectively; Figure S6, Supplementary File S2). At the genera level, *Klebsiella, Citrobacter, Veillonella,* and *Enterococcus* were more abundant in neonates exposed to formula milk, while *Staphylococcus, Parabacteroides, Phocaeicola,* and *Bacteroides* were less abundant (*p*-values 0.001, <0.001, 0.012, 0.029, <0.001, <0.0001, <0.001, and <0.001, respectively; Figure S7, Supplementary File S2). Differently abundant species between neonates who did and did not receive formula milk in the first 7 days of life are detailed in Figure S8, Supplementary File S2.

Neonates of mothers who took probiotics during pregnancy had a lower abundance of Pasteurellaceae and Streptococcaceae, but a higher abundance of Bacteroidaceae (*p*-values 0.031, 0.014, and 0.035; Figure S6, Supplementary File S7). These neonates also had a lower abundance of *Haemophilus* and *Streptococcus* (*p*-values 0.045 and 0.011; Figure S7, Supplementary File S2) [*Streptococcus vestibularis* and *Streptococcus thermophilus* (*p*-values 0.032 and 0.028; Figure S8, Supplementary File S2)]. Neonates of mothers who received antibiotics during the last trimester of pregnancy had a higher abundance of Enterococcaceae (*p*-value 0.043) [*Enterococcus faecalis* (*p*-value 0.017, respectively; Figure S6 and Figure S8, Supplementary File S2)] and a higher abundance of *Streptococcus parasanguinis* (*p*-value 0.015, Figure S8, Supplementary File S2). Neonates of mothers who received intrapartum antibiotics had a lower abundance of Actinobacteria (*p*-value <0.0001; Figure S5, Supplementary File S2). These neonates had a higher abundance of Pasteurellaceae (*Haemophilus*) and a lower abundance of Bifidobacteriaceae (*Bifidobacterium*) (*p*-values 0.002, 0.001, <0.0001, and <0.0001, respectively; Figures S6, S7, Supplementary File S2). Furthermore, a higher abundance of *Haemophilus parainfluenzae* (*p*-values <0.001; Figure S8, Supplementary File S2) was found in neonates whose mothers received antibiotics during delivery.

### Antibiotic resistance genes

Overall, 913 ARGs belonging to 27 classes were identified. The most abundant ARGs were *tetQ, tetM, tetW, tetK* (tetracycline)*, qacD, qacC, qacA* (quaternary ammonium compound), *sitABCD* (peroxide), *msrD, msrA* (macrolide-streptogramin-B), *ermF, ermB, ermC* (macrolide-lincosamide-streptogramin-B), *mdfA* (macrolide-aminoglycoside-tetracycline-quinolone-amphenicol-rifamycin), *mefA* (macrolide), *lsaA* (lincosamide-streptogramin-A), *fosB* (fosfomycin), *sul2* (folate pathway antagonist), *blaZ*; and *mecA* (beta-lactam).

The abundance of ARGs correlated with the bacterial species composition (Mantel test, statistic r 0.46, *p*-value 0.001). Spearman correlation analyses between the relative abundance of bacterial genera and species and ARGs can be found in Figures S9, S10, Supplementary File S2).

There was an association between gestational age, birth weight, mode of delivery, formula milk and the overall abundance of ARGs (PERMANOVA test, *p*-values <0.05; Table 2). There was no association between sex, ethnicity, probiotic use during pregnancy, antibiotic use during the last trimester of pregnancy, intrapartum antibiotic use, and the overall abundance of ARGs. A summary of the clinical factors associated with differences in the intestinal resistome can be found in Table 3.

**Table 3:** Summary on the association between clinical factors and the neonatal intestinal resistome.

Analysis of resistance genes	Delivery mode	Formula milk in first 7 days of life	Probiotics during pregnancy (any trimester)	Antibiotics during pregnancy (last trimester)	Intrapartum antibiotics
**Principal coordinate analyses**	Clustered apart	Clustered apart	No difference	No difference	No difference
**Richness and diversity**	No difference	No difference	No difference	No difference	No difference
**Overall abundance**	No difference	No difference	No difference	Higher in neonates whose mothers took antibiotics	No difference
**Number of ARGs differently abundant**	119	43	33	36	37

### The effect of delivery mode on the abundance of ARGs

**Figure 1:**
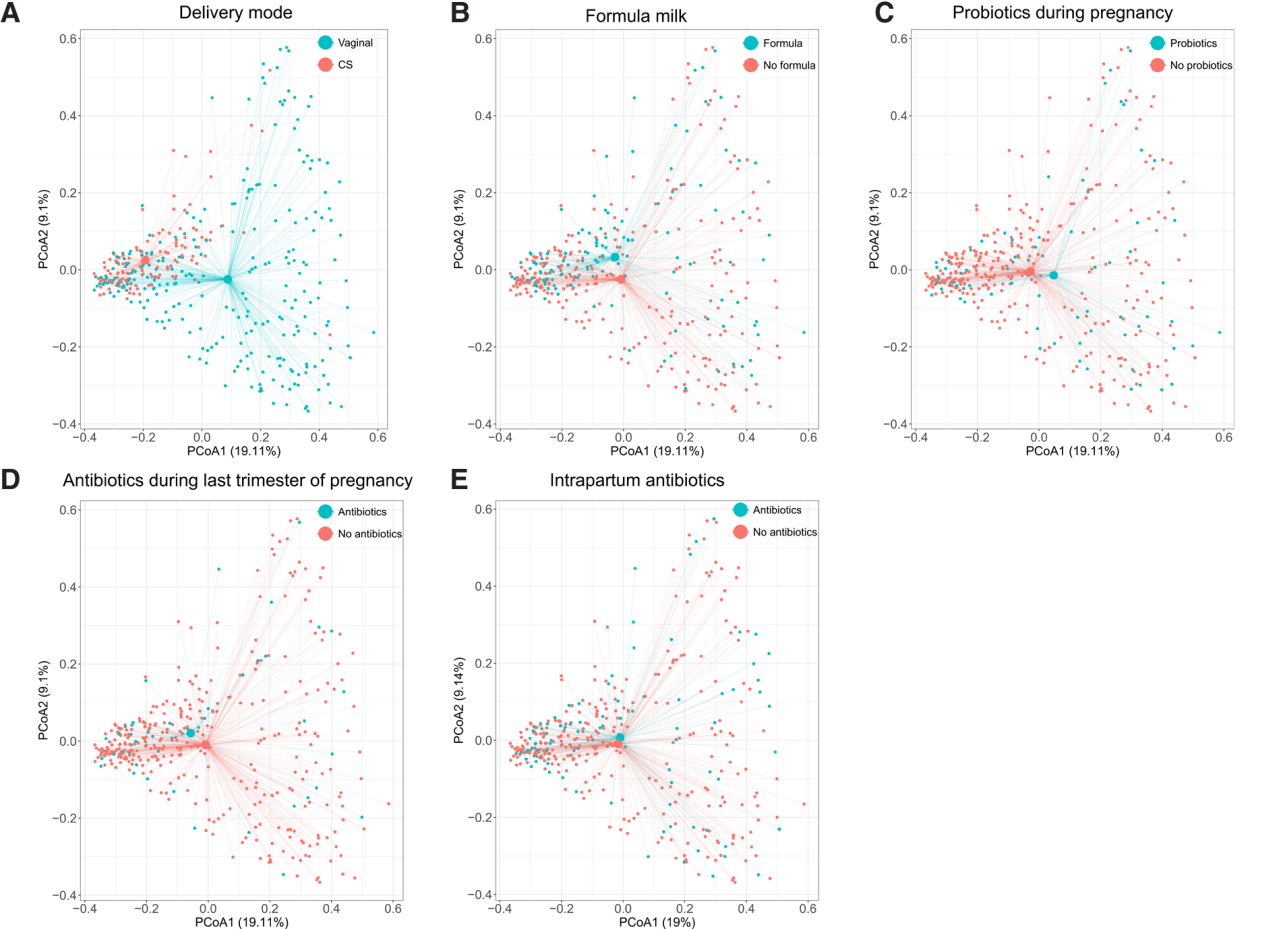
Principal coordinate analyses of resistome profiles done on the normalised read counts of 913 ARGs detected in 390 infants. Axes indicate the percentage of total variance. Colours indicate delivery mode (**A**), formula milk during the first 7 days of life (**B**), probiotics use during pregnancy (any trimester) (**C**), antibiotics use during pregnancy (last trimester) (**D**), and intrapartum antibiotic use (**E**). The centroids indicate average resistome composition. Lines connect centroids to corresponding data points. ARGs, antibiotic resistance genes; CS, Caesarean section.

In the PCoAs, resistome profiles of neonates born by CS and those born vaginally clustered apart (Figure 1). There was no difference in ARGs richness or diversity (Figure S11, Supplementary File S2) or overall abundance of ARGs (median of total normalised ARG counts, 531, IQR: 315–1,025 vs. 636, 413–1,011, *p*-value 0.28).

**Figure 2:**
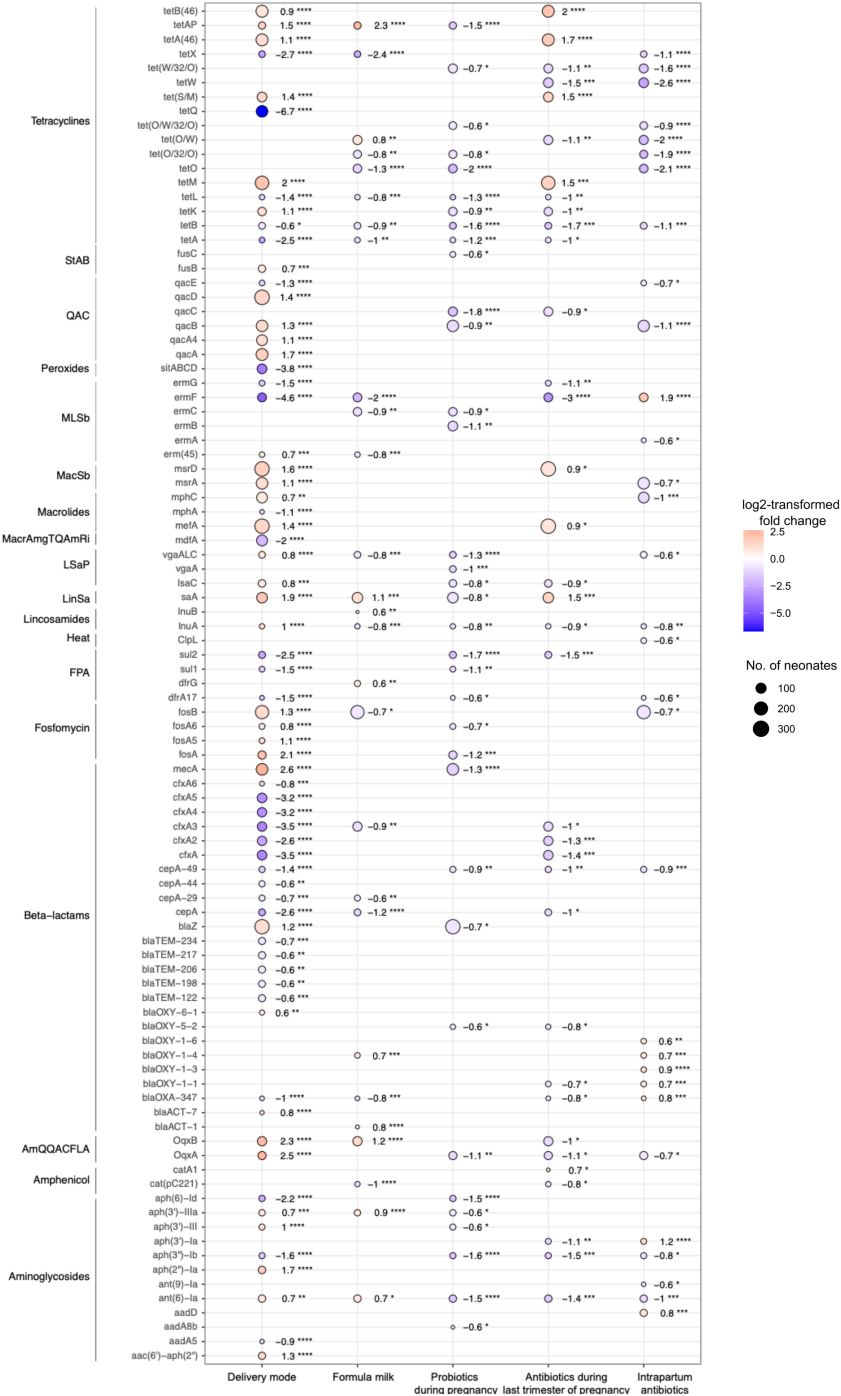
Differentially abundant antibiotic resistance genes. Dots depict genes associated with an FDR-corrected *p*-value of less than 0.05 and linear fold-change of at least 1.5 in the DESeq2 analyses. Colours of the dots represent the log_2_FC and size of the dots the number of neonates in whom ARGs were detected. Red colour and positive values of log_2_FC indicate the gene is more abundant in neonates born by Caesarean section, who received formula milk in the first 7 days of life, or whose mothers took probiotics (any trimester) or antibiotics during pregnancy (last trimester) or received intrapartum antibiotics. Blue colour and negative value of log_2_FC indicate that the gene is more abundant in neonates born vaginally, who were exclusively breastfed in the first 7 days of life or whose mothers did not take probiotics (any trimester) or antibiotics during pregnancy (last trimester) or did not receive intrapartum antibiotics. Wilcoxon rank sum test: *p < 0.05; **p < 0.01; ***p < 0.001; ****p < 0.0001. log_2_FC, log_2_-transformed fold-change; StAB, steroid antibacterial; QAC, quaternary ammonium compounds; MLSb, macrolide, lincosamide, streptogramin-B; MacSb, macrolide, streptogramin-B; MacrAmgTQAmRi, macrolide, aminoglycoside, tetracycline, quinolone, amphenicol, rifamycin; LSaP, lincosamide, streptogramin-A, pleuromutilin; LinSa, lincosamide, streptogramin-A; FPA, folate pathway antagonist; AmQQACFLA, amphenicol, quinolone, quaternary ammonium compounds, folate pathway antagonist.

In total, 119 ARGs were differentially abundant between the two groups (DESeq2, FDR-corrected *p*-value <0.05): *msrD, qacA, lsaA, tetM*, and *mecA* were more than three-fold more abundant in neonates born by CS; *mdfA, cfxA5, cfxA, sitABCD*, and *tetQ* were more than three-fold more abundant in neonates born vaginally (Figure 2).

### The effect of formula milk on the abundance of ARGs

Resistome profiles clustered according to the feeding method in the first week of life (Figure 1). There was no difference in richness or diversity of ARGs (Figure S11, Supplementary File S2) or in the overall abundance of ARG between neonates who did or did not receive formula milk during the first week of life (median of total normalised ARG counts 665, IQR: 422–1,026, vs. 575, IQR: 346–1,009, *p*-value 0.3).

Forty-three ARGs were differentially abundant between the two groups (DESeq2, FDR-corrected *p*-value <0.05); two of them were detected in more than 100 neonates. *LsaA* (lincosamide-streptogramin-A) was more abundant in neonates who received formula milk, and *fosB* (fosfomycin) was more abundant in neonates who were exclusively breastfed (Figure 2).

### The effect of probiotic use during pregnancy on the abundance of ARGs

Resistome profiles did not cluster according to probiotic use in pregnancy (Figure 1), and there was no difference in ARG richness and diversity between neonates of mothers who took probiotics during pregnancy and those who did not (Figure S11, Supplementary File S2) or in the overall abundance of ARGs (medians of total normalised ARG counts 609, IQR: 375–992, vs. 685, IQR: 325–1,030, respectively; *p*-value 0.7).

Thirty-five ARGs were differentially abundant between the two groups (DESeq2, FDR-corrected *p*-value <0.05). Five ARGs were detected in more than 100 neonates. *BlaZ, mecA* (beta-lactam), *qacB* (quaternary ammonium compounds), *ermB* (macrolide-lincosamide-streptogramin-B), and *lsaA* (lincosamide-streptogramin-A) were less abundant in neonates of mothers who took probiotics during pregnancy (Figure 2).

### The effect of antibiotic use during the last trimester of pregnancy on the abundance of ARGs

Resistome profiles did not cluster according to antibiotic use in the last trimester of pregnancy (Figure 1). There was no difference in ARG richness and diversity between neonates of mothers who received antibiotics in the last trimester of pregnancy and these who did not (Figure S11, Supplementary File S2). However, the overall abundance of ARGs was higher in neonates whose mothers took antibiotics (median 833, IQR: 530–1,269 vs. 584, 361–934, *p*-value 0.018).

Thirty-six ARGs were differentially abundant between the two groups (DESeq2, FDR-corrected *p*-value <0.05); nine ARGs were present in more than 100 neonates. In neonates of mothers who did received antibiotics, *msrD* (macrolide-streptogramin-B), *ermB* (macrolide-lincosamide-streptogramin-B), *mefA* (macrolide), *lsaA* (lincosamide-streptogramin-A), and *tet(S/M)*, *tet(M)*, *tetA(46)*, *tetB(46)* (tetracycline) were more abundant. In neonates whose mothers did not receive antibiotics *cfxA* (beta-lactam) and *tetW* (tetracycline) were more abundant (Figure 2).

### The effect of intrapartum antibiotic use on the abundance of ARGs

Resistome profiles did not cluster according to intrapartum antibiotic use (Figure 1), and intrapartum antibiotic use did not influence ARGs’ richness and diversity (Figure S11, Supplementary File S2). There was also no difference in the overall number of ARGs between neonates of mothers who received intrapartum antibiotics and those who did not (medians of total normalised counts 642, IQR: 378–1,043, vs. IQR: 609, 370–984, *p*-value 0.7).

Thirty-seven ARGs were differentially abundant between neonates whose mothers did or did not receive intrapartum antibiotics (DESeq2, FDR-corrected *p*-value <0.05). Five of these ARGs were present in more than 100 neonates. The abundance of *tetW* (tetracycline)*, qacB*, *qacA4* (quaternary ammonium compounds)*, mphC* (macrolide)*, msrA* (macrolide-streptogramin-B), and *fosB* (fosfomycin) was higher in neonates whose mothers did not receive intrapartum antibiotics (Figure 2).

## Discussion

In this study, the largest to date to use shotgun metagenomic sequencing to investigate the neonatal resistome, the neonatal intestine was found to harbour a high abundance and variety of ARGs, even in the absence of direct antibiotic exposure. Our findings are consistent with previous studies, which also found that the neonatal intestine harbours a high abundance of ARGs and that the most commonly found ARGs in neonates confer resistance to aminoglycosides, beta-lactams, macrolides, tetracyclines, or multidrug resistance (25). Similar to other studies, we found a strong association between the bacterial species composition of the intestinal microbiome and the composition of the resistome (20, 26–30). Previously, ARGs in neonates have been most frequently associated with the orders Bacteroidales and Enterobacteriales and the genera *Bacteroides, Clostridioides, Escherichia*, and *Staphylococcus* (26–30). An inverse correlation between the abundance of *Bifidobacterium* and the abundance of ARGs has been reported (26, 31). As in previous studies (32), in our cohort, neonates born by CS and neonates of mothers who received intrapartum antibiotics were found to have a significantly lower abundance of *Bifidobacterium*. Bifidobacteria are not only associated with a lower abundance of ARGs but also other health benefits, such as lower rates of allergic sensitisation, eczema, or asthma (33) and higher responses to vaccines (34).

A further factor that influenced the abundance and type of ARGs in the neonatal intestine was delivery mode. This is likely because delivery mode has a strong effect on the phylogenetic composition of the intestinal microbiome. Vertical transmission of ARGs from mothers to neonates occurs through contact, either during delivery with the genital tract or later through contact with the nasopharyngeal, oral, and skin microbiome (26, 35–37). Neonates born vaginally are, therefore, likely colonised by ARGs from the maternal vaginal and intestinal microbiome, whereas infants born by CS predominantly acquire ARGs from the maternal skin microbiome (4). As an example, in our cohort and in previous studies, a higher abundance of *mecA* was found in neonates born by CS (38, 39), which is likely attributable to colonisation by *Streptococcus* and *Staphylococcus* from the skin microbiome (4). Previous studies have reported a higher overall abundance of ARGs and a higher abundance of multidrug resistance-conferring genes *bcr, mls_abc, mls_hdr, macAB, ykk*, and *mdr*; the tetracycline resistance-conferring gene *tet_efflux*; the chloramphenicol resistance-conferring gene *blt*; and the vancomycin resistance-conferring gene *vanA* in the stool of neonates born by CS (39–41). A higher abundance of the beta-lactam resistance-conferring gene *bla*_A_, tetracycline resistance-conferring gene *tet_RPP*, and multidrug resistance-conferring genes *mexEF* and *mexHI* has been found in stool of vaginal-born neonates (41). Delivery mode has been reported to affect the composition of the intestinal microbiome for up to 6–12 months (42–46), for *Clostridia* spp. even up to seven years of age (47). Therefore, the association between delivery mode and differences in the composition of intestinal resistome might be clinically relevant, as the first year of life is the period with the highest risk for bacterial infection in children. However, longitudinal studies are needed to investigate how long these differences in the resistome persist. Some previous studies did not find an influence of delivery mode on the abundance of ARGs in neonatal stool, but this might have been because they were underpowered (29, 48–50). Furthermore, previous studies that used shotgun metagenomic sequencing to investigate the intestinal resistome in neonates often included neonates who were treated with antibiotics or for whom antibiotic exposure was not specified (26–27, 29, 35, 41, 48–54).

Consistent with our findings, it has previous been reported that breastfeeding influences the abundance of ARGs in the neonatal intestine (49). Also, similar to our findings, infant sex has previously been reported to have little effect on the abundance of ARGs in neonates (25). None of the previous studies investigated the effect of gestational age, birth weight, ethnicity, and probiotic use during pregnancy on the neonatal resistome. There have only been two previous studies that investigated whether maternal antibiotic use during pregnancy influences the neonatal intestinal resistome. One study did not find an association (54), and the other reported a higher abundance of *bla*_cepA_ and *bla*_cfxA_ in stool of neonates whose mothers were exposed to antibiotics (48). As in our study, it has been reported that intrapartum antibiotics does not affect the abundance of ARGs in neonatal stool (29, 49). However, some studies did find a higher abundance of ARGs in the neonatal intestinal microbiome (conferring resistance to beta-lactams, aminoglycosides, and fosfomycin) (26, 54, 55).

While the abundance of ARGs between neonates and their mothers was not compared in this study, previous studies have reported a higher abundance of ARGs in the intestine of neonates compared with their mothers (26, 35, 36, 39, 40). Again, this is likely explained by the bacterial phylogenetic composition. Neonates have a high abundance of Gammaproteobacteria, including Enterobacteriaceae, in their intestinal microbiome, which carry high numbers of ARGs (2, 26, 56–58).

The strengths of this study include the use of shotgun metagenomic sequencing that allowed simultaneous investigation of bacterial and resistome composition, the large sample size, and the absence of direct antibiotic exposure. A limitation of metagenomic sequencing is that it does not allow differentiation between genotypic and phenotypic resistance. It is, therefore, not possible to determine if the identified ARGs are expressed. Other limitations of this study include the absence of a longitudinal follow-up, the use of only one database for ARGs identification, the lack of detailed investigation of bacteriophages due to the absence of enrichment for viruses prior to extraction, and non-performance of RNA sequencing. Furthermore, although it has previously been reported that in neonates direct antibiotic use is associated with a higher abundance of ARGs and probiotic use with a lower abundance (25), this was not investigated in this study due to low numbers.

## Conclusion

In this study, phylogenetic composition was strongly associated with the resistome composition. Other factors that were associated with the abundance of ARGs were delivery mode, gestational age, birth weight, feeding method, and antibiotics in the last trimester of pregnancy. Sex, ethnicity, probiotic use during pregnancy, and intrapartum antibiotics had little effect on the abundance of ARGs.

The neonatal intestine has a low colonisation resistance, which potentially makes it easier for antibiotic-resistant populations to establish themselves. In addition, neonates have a high abundance of Gammaproteobacteria*,* which are associated with high rates of horizontal gene transfer (59). Future studies will help identify the clinical relevance of the factors that are associated with differences in the intestinal resistome and in the development of evidence-based interventions to modulate the abundance of ARGs in neonates, for example, by the use of prebiotics, probiotics, and bacteriophages.

## Data Availability

Quality-filtered non-human read FASTQ files were deposited in the European Nucleotide Archive under the project PRJEB55714. Codes used for the pre-processing of sequencing reads (quality-filtering, removal of human DNA, etc.) are available on Github (https://github.com/miraclelab-unifr/Eczema).

## Supplementary material

Supplementary File 1 R code

Supplementary File 2 Supplementary figures and tables

Supplementary File 3 Distribution of log2-transformed normalised reads of ARGs

Supplementary Table 1 Excel table containing results of DESeq2 analyses

Supplementary Table 2 Excel table containing results of Wilcoxon tests on phyla

Supplementary Table 3 Excel table containing results of Wilcoxon tests on families

Supplementary Table 4 Excel table containing results of Wilcoxon tests on genera

Supplementary Table 5 Excel table containing results of Wilcoxon tests on species

Supplementary Table 6 Bacteria and viruses identified with Bracken v2.6.2

Supplementary Table 7 ARGs identified with ResFinder gene database v4

Supplementary Table 8 Metadata
