# Supplementary material for: Metagenomics analysis of the neonatal intestinal resistome
Source: Front Pediatr. 2023 Jun 16;11:1169651. doi: 10.3389/fped.2023.1169651 (PMC10313230; doi:10.3389/fped.2023.1169651)
Supplement: Supplementary File 2 — Supplementary figures and tables [file Datasheet2.pdf]

Supplementary Figure 1. Principal coordinate plots based on species abundance.

Species microbiome profiles analysed by the Kraken2/Bracken pipeline according to delivery mode (A.), formula milk during the first 7 days of life (B.), probiotic use during pregnancy (any trimester) (C.), antibiotics use during pregnancy (last trimester) (D.) and intrapartum antibiotic use (E.). Principal coordinate analyses (PCoAs) were performed on the relative abundance of 2803 species detected in 390 neonates. Axes report the total variance. For intrapartum antibiotic use, participants with missing values were excluded from the plot (E.) which was computed on 99.5% (388/390) samples and 99.9% (2802/2803) species. Centroids are connected to corresponding data points by lines.

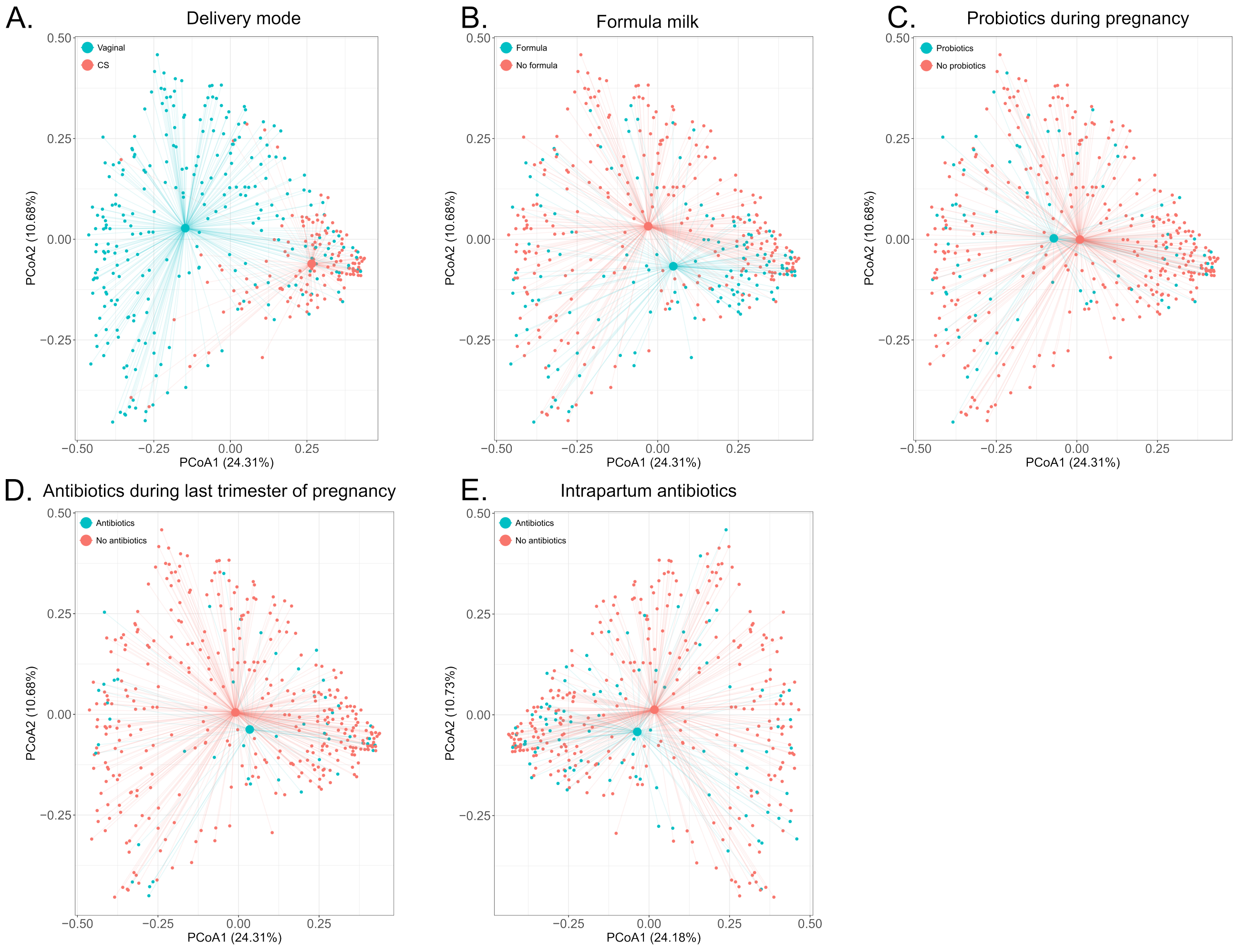

CS - Caesarean section

Supplementary Figure 2. Principal coordinate plots based on genera abundance.

Same as Supplementary Figure 1, except that results from the microbiome profiles are based on the 881 genera identified by the Kraken2/Bracken pipeline.

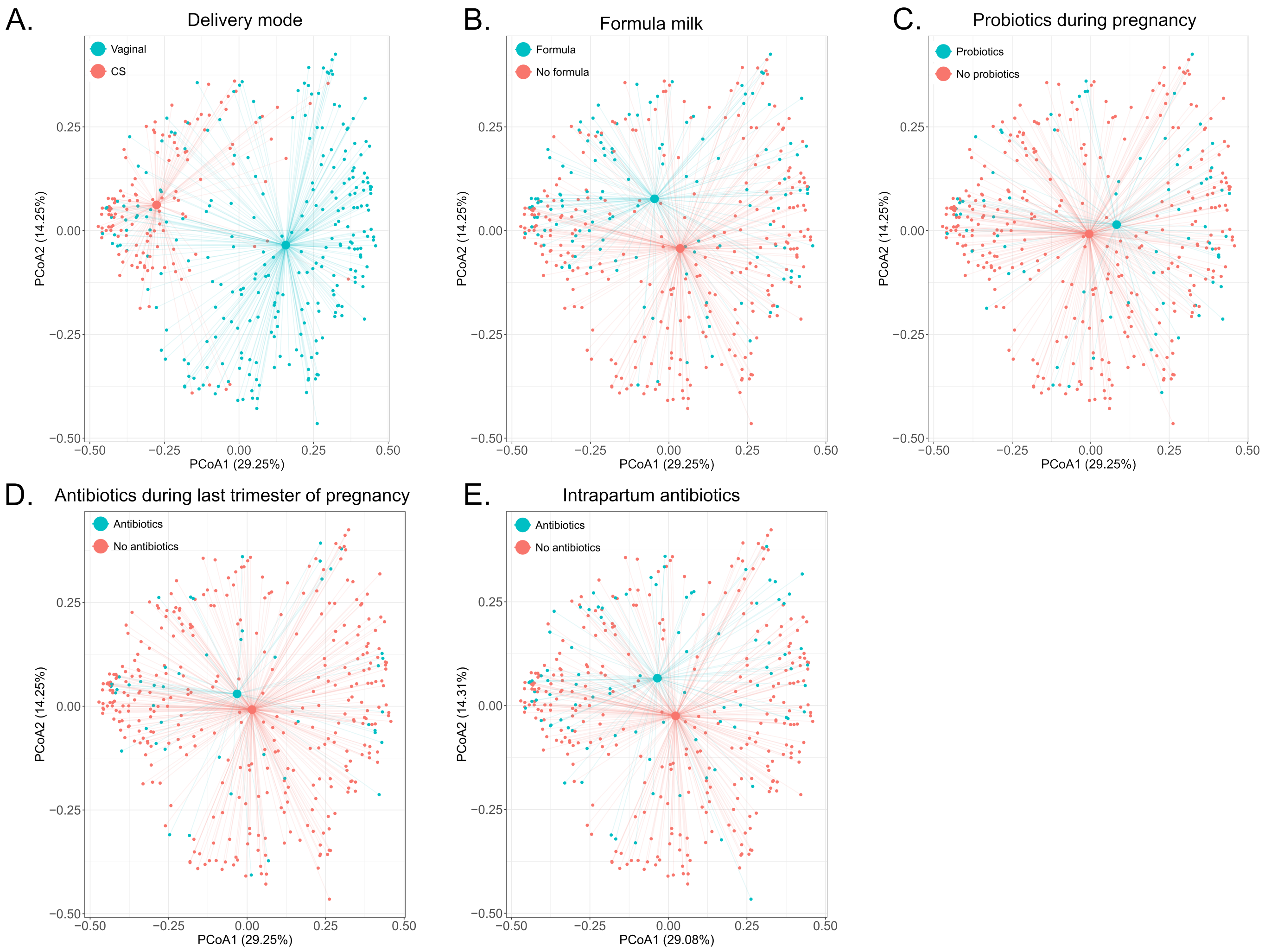

CS - Caesarean section

Supplementary Figure 3. Ecological indices.

Richness, Shannon, and Simpson diversity were computed on species identified by Kraken2/Bracken.

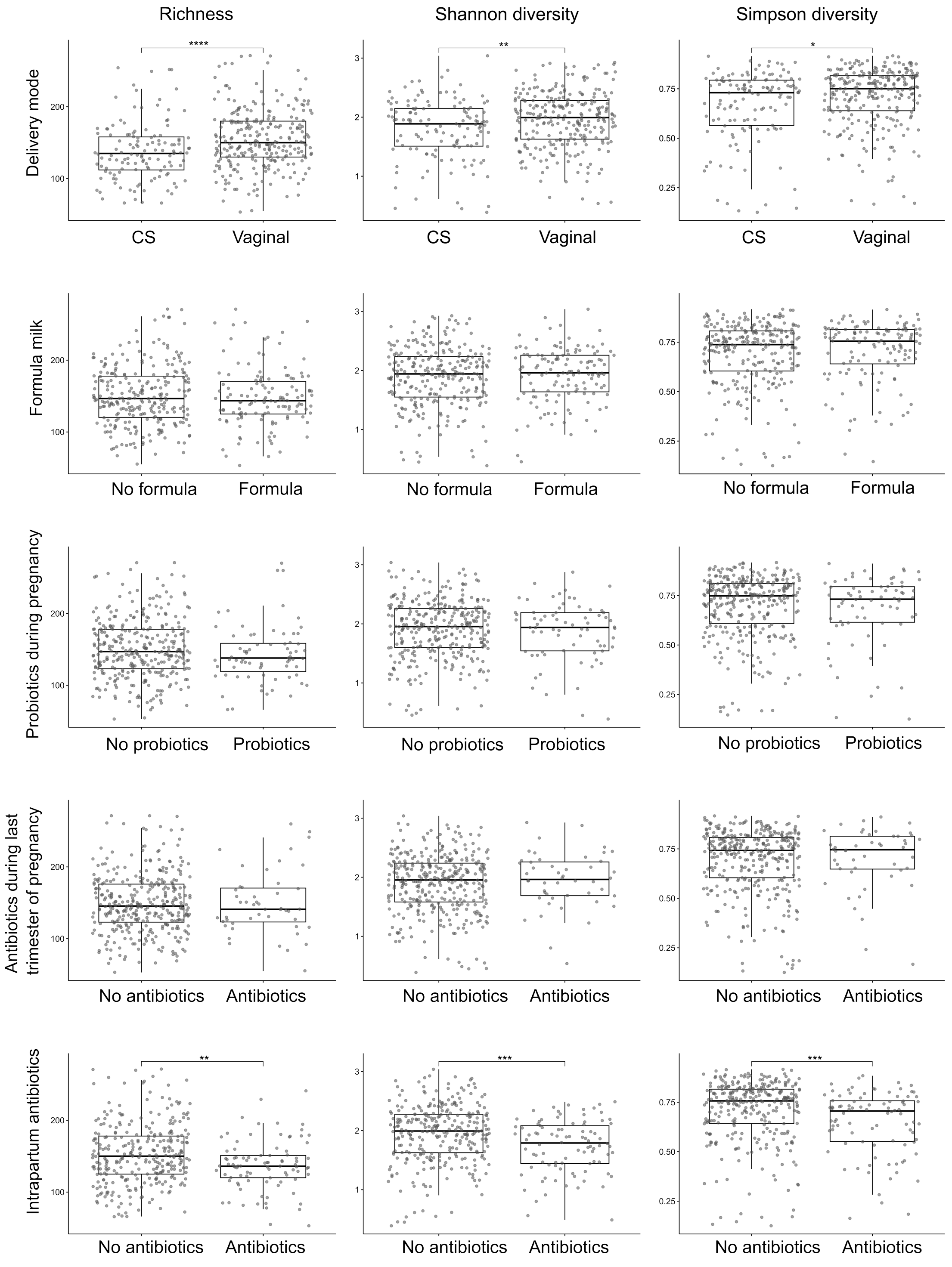

Wilcoxon rank sum test, *p < 0.05; **p < 0.01; ***p < 0.001; ****p < 0.0001

CS - Caesarean section

Supplementary Figure 4. Bacteriophages composition.

Normalised counts mapping to bacteriophages after square-root transformation. Wilcoxon tests were computed on non-transformed values.

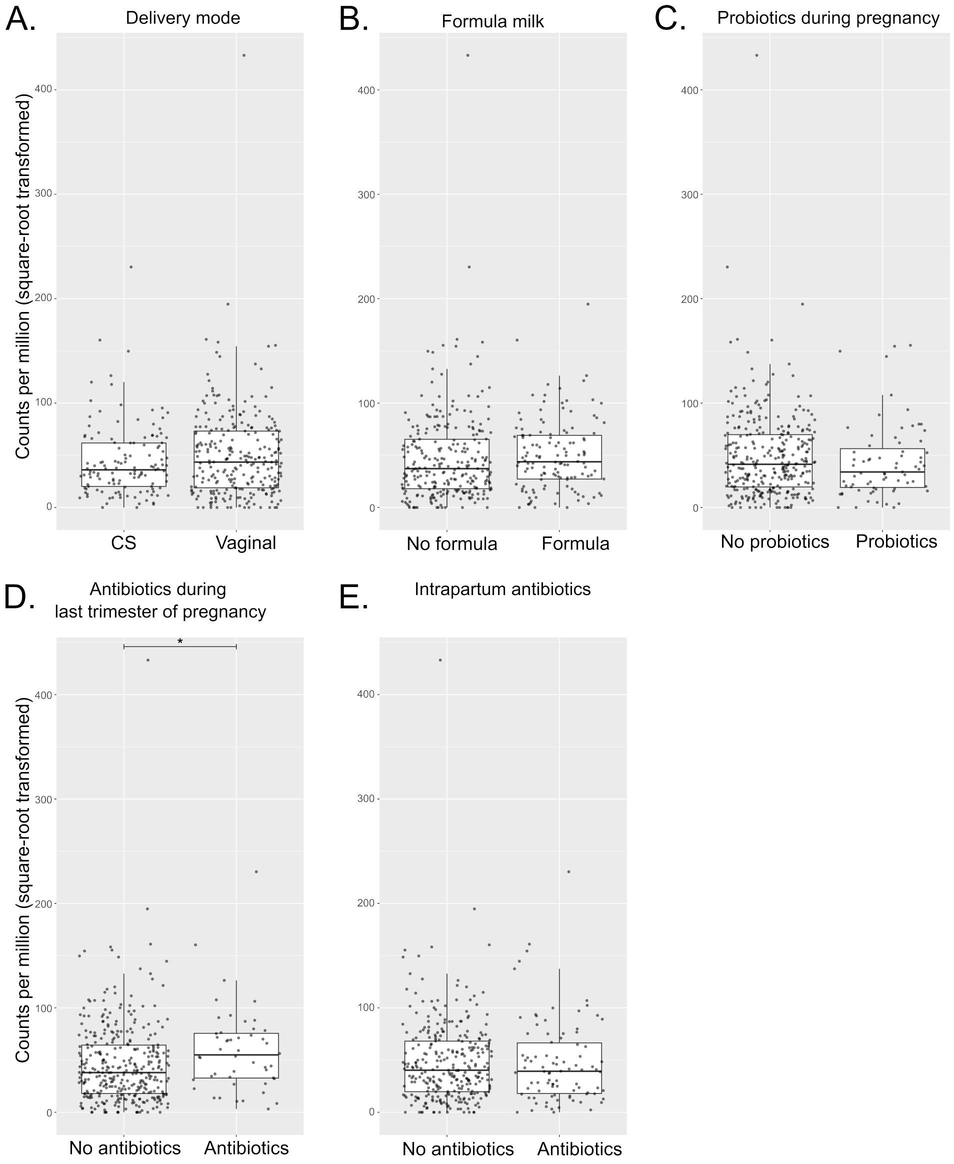

Wilcoxon rank sum test, *p ≤ 0.05

CS - Caesarean section

Supplementary Figure 5. Differentially abundant phyla.

Selected phyla associated with a Wilcoxon-rank-sum p-value of less than 0.05 and a linear fold-change of at least 1.5 in at least one analysis. Colours of the dots represent the log2-transformed fold-change (log2FC) and size of the dots in how many neonates the phyla were detected. Red colour and positive values of log2FC indicate the gene is more abundant in neonates born by Caesarean section, who received formula milk in the first 7 days of life, or whose mothers took probiotics (any trimester) or antibiotics during pregnancy (last trimester) or received intrapartum antibiotics. Blue colour and negative value of log2FC indicate that the gene is more abundant in neonates born vaginally, who were exclusively breastfed in the first 7 days of life or whose mothers did not take probiotics (any trimester) or antibiotics during pregnancy (last trimester) or did not receive intrapartum antibiotics.

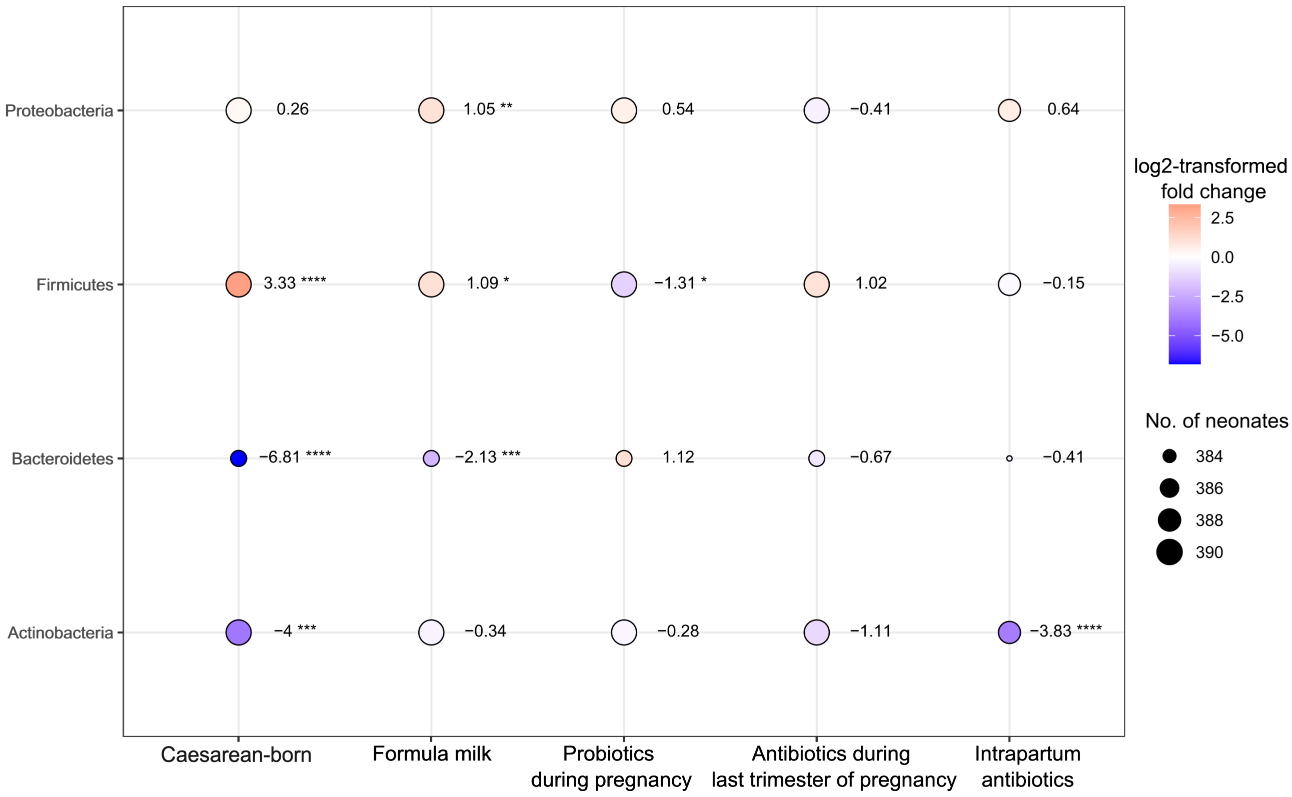

Wilcoxon rank sum test, *p < 0.05; ** p < 0.01; ***p < 0.001; ****p < 0.0001

Supplementary Figure 6. Differentially abundant families.

Same as Supplementary Figure 5 except that results are from analyses at the family level.

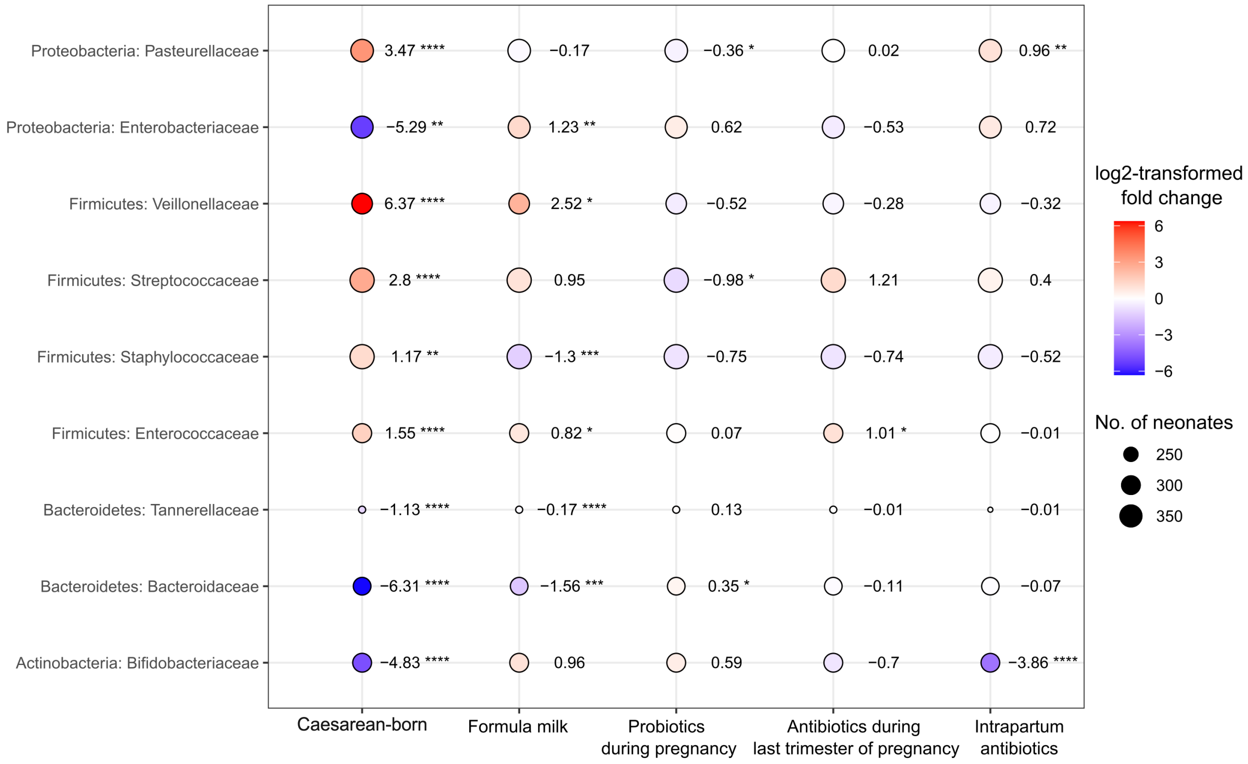

Wilcoxon rank sum test, *p < 0.05; ** p < 0.01; ***p < 0.001; ****p < 0.000

Supplementary Figure 7. Differentially abundant genera.

Same as Supplementary Figure 5, except that results are from analyses at the genus level.

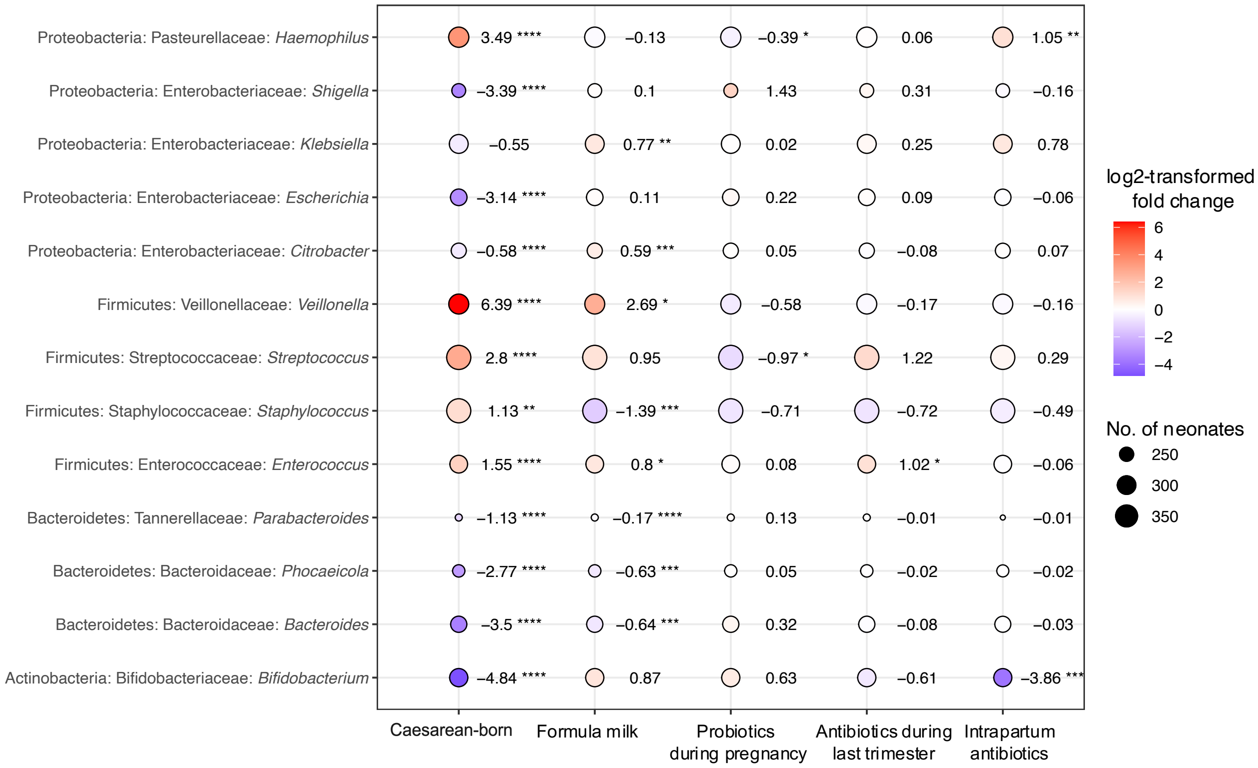

Wilcoxon rank sum test, *p < 0.05; ** p < 0.01; ***p < 0.001; ****p < 0.0001

Supplementary Figure 8. Differentially abundant species.

Same as Supplementary Figure 5, except that results are from analyses at the species level.

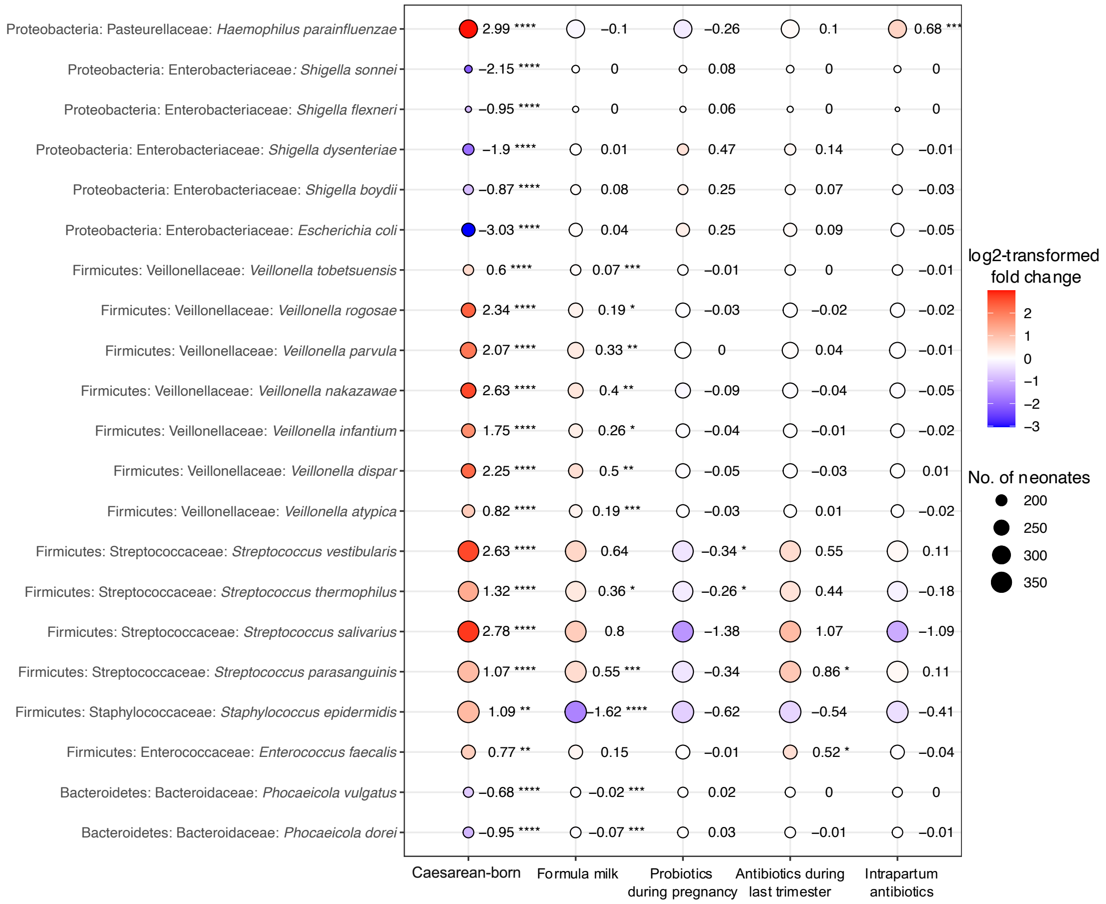

Wilcoxon rank sum test, *p < 0.05; ** p < 0.01; ***p < 0.001; ****p < 0.0001

Supplementary Figure 9. Spearman correlation analyses between the relative abundance of bacterial genera and ARGs.

Fourteen bacteria genera with a relative abundance of ≥ 0.5 in at least 50 samples were selected. Forty-seven ARGs with normalised counts ≥ 0.1 in at least 50 samples were also selected for the analyses. Correlation was performed in R with the function cor (method ="spearman", use = "complete.obs"). Correlation values are colour-scaled according to the legend reported on the left (red = positive association; blue= negative association). Plotting was done with the function ggheatmap from the heatmaply R package v 1.4.2. The name of the genera is reported with the corresponding taxonomy id. Please note that the figure only shows a correlation between the abundance of genera and ARGs and not a direct link between certain genera and ARGs (e.g. the ARGs are not necessary present in the genera with high correlation).

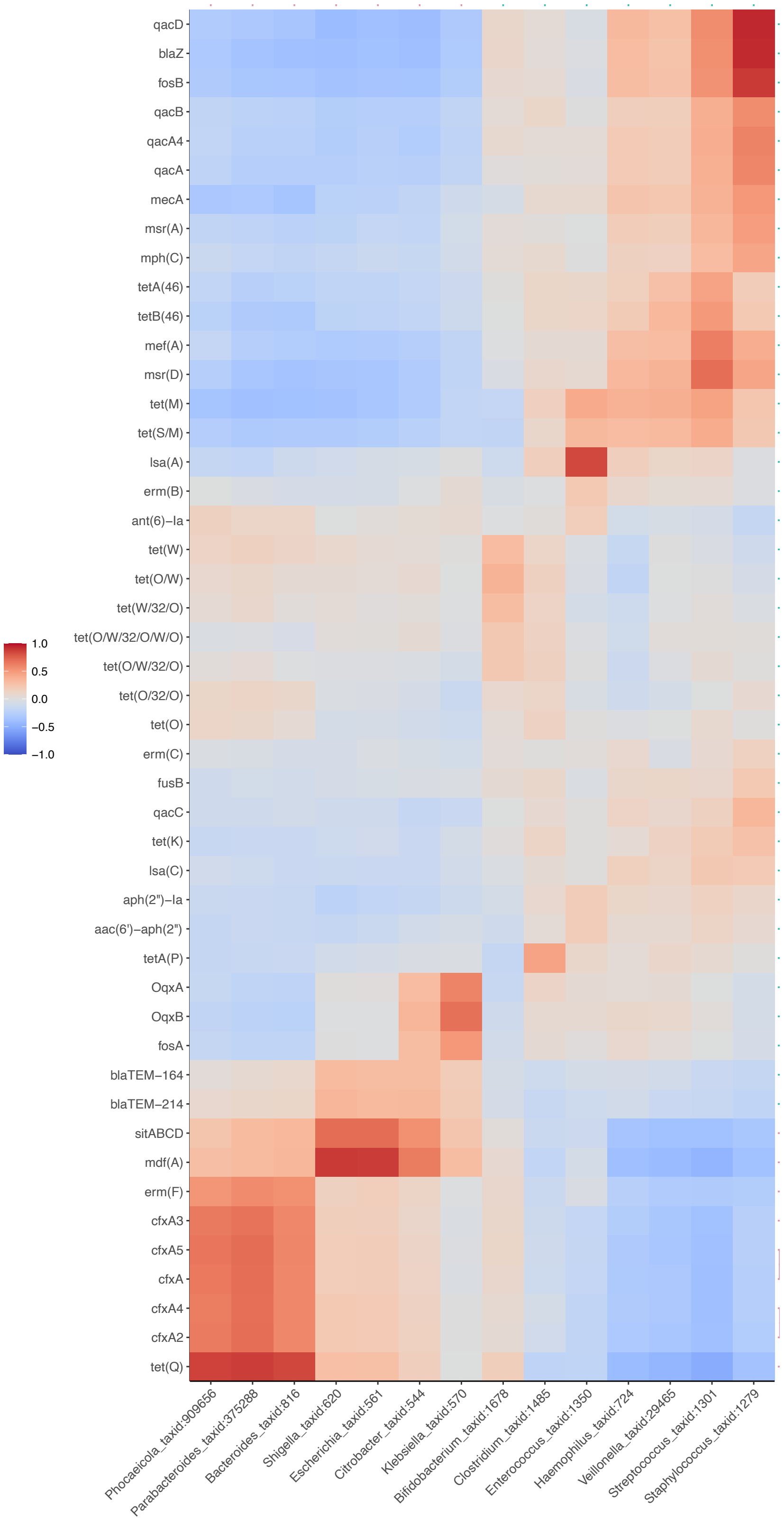

Supplementary Figure 10. Spearman correlation analyses between the relative abundance of bacterial species and ARGs.

Twenty-seven bacteria species with a relative abundance of ≥ 0.5 in at least 50 samples were selected. Forty-seven ARGs with normalised counts ≥ 0.1 in at least 50 samples were also selected for the analyses. Correlation was performed in R with the function cor (method ="spearman", use = "complete.obs"). Correlation values are color-scaled according to the legend reported on the left (red = positive association; blue= negative association). Plotting was done with the function ggheatmap from the heatmaply R package v 1.4.2. The name of the species is reported with the corresponding taxonomy id. Please note that the figure only shows a correlation between the abundance of species and ARGs and not a direct link between certain species and ARGs (e.g. the ARGs are not necessary present in the species with high correlation).

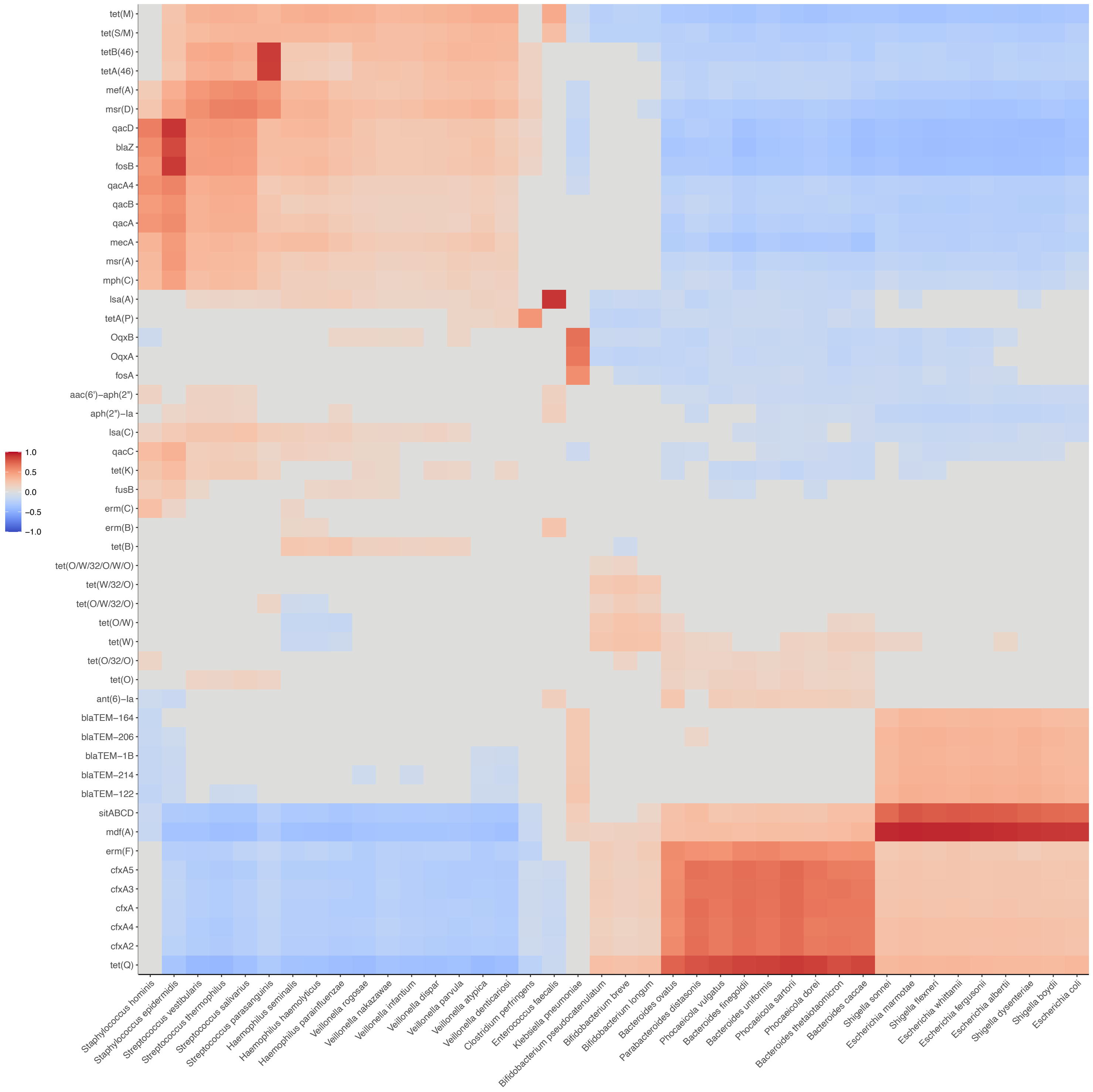

Supplementary Figure 11. ARGs richness and diversity.

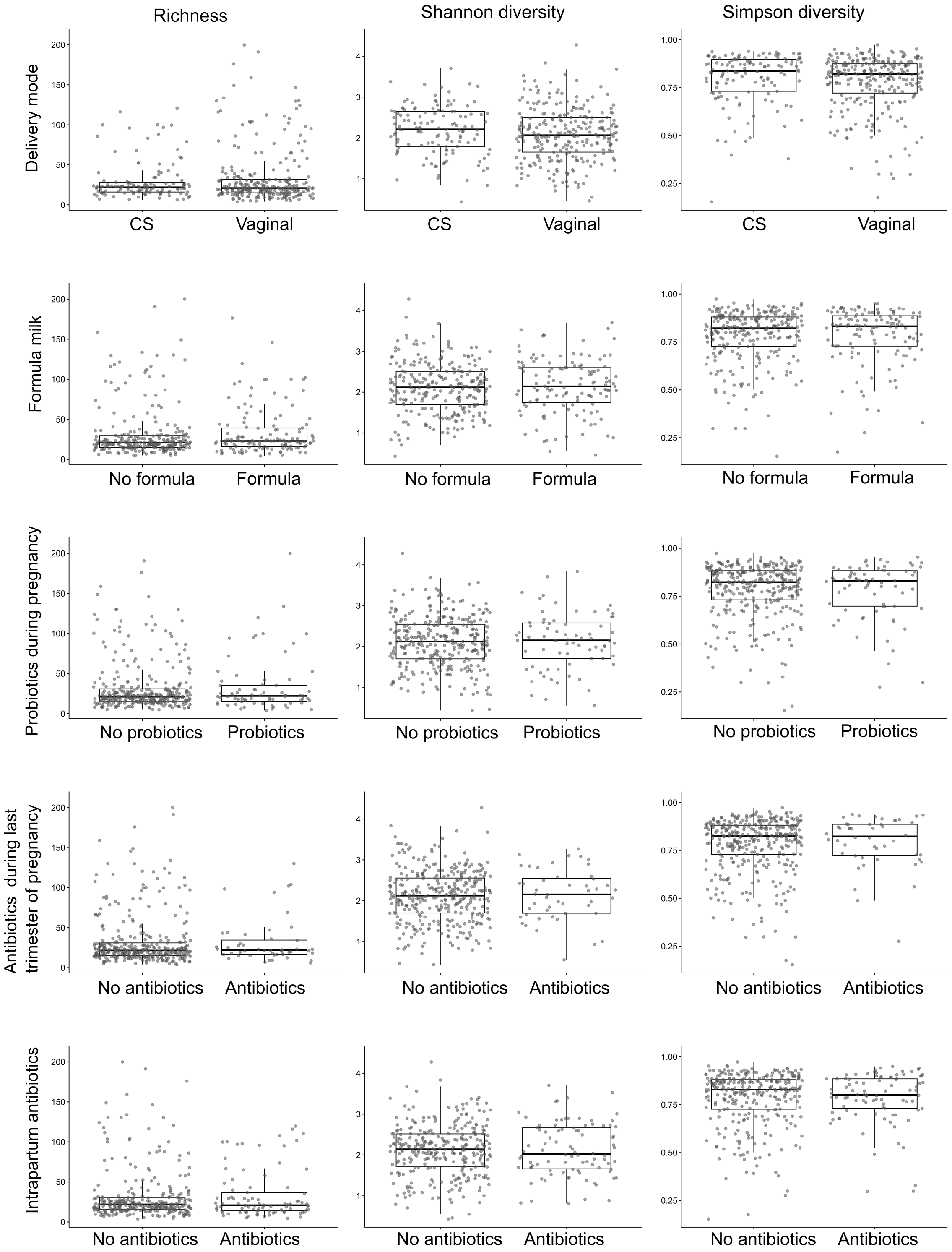

CS - Caesarean section
