# Supplementary material for: Metagenomics analysis of the neonatal intestinal resistome
Source: Front Pediatr. 2023 Jun 16;11:1169651. doi: 10.3389/fped.2023.1169651 (PMC10313230; doi:10.3389/fped.2023.1169651)
Supplement: Supplementary File 3 — Distribution of log2-transformed normalised reads of ARGs [file Datasheet3.pdf]

Supplementary file 3: Distribution of log2-transformed normalised reads mapping to a given ARG in each of the 390 samples. Plots are grouped in columns (according to variables) and rows (ARGs).

Y-axis reports the log2-transformed normalised counts whereas x-axis the 390 samples. Therefore, each bar corresponds to the log2-transformed number of normalised reads mapping to a given ARG in a given sample. Samples are grouped and coloured according to the terms of comparisons (c-section vs vaginal for delivery mode; yes = formula in first 7 days of life, probiotics during pregnancy (any trimester), antibiotics during pregnancy (last trimester) and intrapartum antibiotics

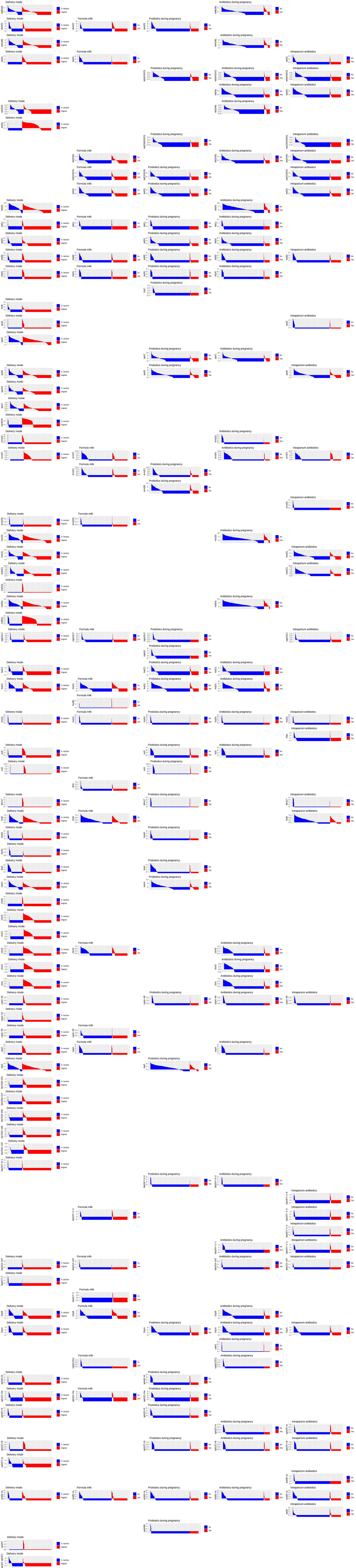

Delivery mode
Formula milk
Probiotics during pregnancy
Antibiotics during pregnancy
Intrapartum antibiotics
C-section
Vaginal
no
yes
No
Yes
