# Supplementary material for: Metagenomics analysis of the neonatal intestinal resistome
Source: Front Pediatr. 2023 Jun 16;11:1169651. doi: 10.3389/fped.2023.1169651 (PMC10313230; doi:10.3389/fped.2023.1169651)
Supplement: Supplementary File 1 — R code [file Datasheet1.pdf]

# Supplementary_materials_software_misbair_resistome

2023-01-11

## Load R libraries, set working directory, read tables

For simplicity, we give information for species analyses. The coding for the other taxonomic levels is the same.

```
#load libraries
library(vegan)
library(DESeq2)
library(openxlsx)
library(ggplot2)

#setwd
#setwd("/data/")

#read table from Kraken2/Bracken
species_bracken = read.delim("bac_vir_fung_counts_bracken.csv", header =TRUE, sep =",")
#rearrange table and correct sampleID so that it matches the metadata
colnames(species_bracken)=  gsub("Counts_no_human_", "", colnames(species_bracken))
colnames(species_bracken) = gsub("\\.", "-", colnames(species_bracken))

#keep only bacteria
species_bracken = species_bracken[grep("d__Bacteria", species_bracken$Taxonomy),]

#label rownames with species name and taxID
rownames(species_bracken) = paste(as.character(species_bracken$Species),  as.character(species_bracken$TaxID), se
p ="_&_")
#remove first three columns containg character values
species_bracken = species_bracken[, -which(colnames(species_bracken) %in% c("Species", "TaxID", "Taxonomy"))]

#convert NA counts in 0
species_bracken[is.na(species_bracken)] = 0

#compute relative abundance
species_bracken_prop = apply(species_bracken,2,function(x) (x/sum(x))*100)


#read table ARG counts

only_paired_no_multiple_mapping = read.delim("only_paired_mapped_no_multiple_mapping_reads", header = FALSE, sep
="\t")

mycolnames_files =  read.delim("my_colnames_bam", header = FALSE, sep =" ")
mycolnames_files = as.character(mycolnames_files)

#correct samples label
mycolnames_files = gsub("no_human_", "", mycolnames_files)
mycolnames_files = gsub("_final.sorted.bam", "", mycolnames_files)
mycolnames_files = c("Allele_ID", "pos","length", "Allele_ID2",mycolnames_files )

colnames(only_paired_no_multiple_mapping)=mycolnames_files

#read metadata

metadata = read.xlsx("metadata.xlsx")
metadata = as.data.frame(metadata)

metadata$SampleID = as.character(metadata$SampleID )

#variable used to select samples
metadata$select_correct_resistome= as.character(metadata$select_correct_resistome)

#print table gene counts

create_table_gene_counts = function(x){
    y = x
    y$Gene = as.character(unlist(sapply(y$Allele_ID, function(x){strsplit(x, split ="_")[[1]][1]})))

    #print number of unique genes
    mygenes = unique(y$Gene )
    print(paste("my unique genes are ", length(mygenes), sep =""))

    n.samples = colnames(y)[!colnames(x)%in% c("Gene","Allele_ID","pos","length",
                                                "Allele_ID2")]

    #create a table to collect results
    mytable.final = data.frame(matrix(rep(NA, length(n.samples)+1), nrow=1))
    colnames(mytable.final) = c("Gene",  n.samples)

    #sum counts for each gene
    for(k in 1:length(mygenes)){
      y.k = y[y$Gene == mygenes[k],]

      #if gene contains one allele
      if(nrow(y.k) == 1){
        mytable.k = y.k[,which(!colnames(y.k) %in% c("Allele_ID","pos","length",
                                                "Allele_ID2", "Gene"))]

        mytable.k$Gene =mygenes[k]
        #reorder column
        mytable.k = mytable.k[, c(ncol(mytable.k), 1:(ncol(mytable.k)-1))]

      }else{
        #if gene contains more than one allele
        mytable.k = y.k[,which(!colnames(y.k) %in% c("Allele_ID","pos","length",
                                                "Allele_ID2", "Gene"))]

        mytable.k = apply(mytable.k,2, function(z) sum(z))
        mytable.k = as.data.frame(t(as.data.frame(mytable.k)))
        rownames(mytable.k) = NULL
        mytable.k$Gene =mygenes[k]
        #reorder column
        mytable.k = mytable.k[, c(ncol(mytable.k), 1:(ncol(mytable.k)-1))]
      }

      mytable.final=rbind(mytable.final,mytable.k)
    }
    mytable.final= mytable.final[-1,]
  return(mytable.final)
}

table_gene_counts_only_paired_no_multiple_mapping = create_table_gene_counts(x = only_paired_no_multiple_mapping)
```

```
## [1] "my unique genes are 2534"
```

## Function used to normalise ARG sequence counts according to the sequence length and bacterial reads

The function provides four different outputs: 1. "counts_with_no_zero_samples" = read count table where samples with 0 counts are excluded; 2. "normalized_without_length" = read counts normalised by only sequence length; 3. "normalized_by_also_length" = read counts normalised by sequence length and bacterial counts; 4. "bracken_bacterial_counts" = table containing the bacterial counts used for step 3; 5. "table_allele_length" = table containing the length of each ARG sequence.

```
#function to normalize data

normalize_data = function(x, metadata){

  if(all(x$Allele_ID == x$Allele_ID2)){
    print("Allele_ID and Allele_ID2 contain the same values")
  }else{
    stop("Allele_ID and Allele_ID2 DO NOT contain the same values")
  }

  #create the table containing the info of the length for each allele in Kilobase
  mytab_allele_length = x[, c("Allele_ID", "pos", "length","Allele_ID2")]
  mytab_allele_length$length_kb = mytab_allele_length$length/1000

  #label rownames x
  rownames(x) = x$Allele_ID

  #remove first four columns
  x = x[, !colnames(x) %in% c("Allele_ID", "pos", "length","Allele_ID2")]

  #remove samples that have 0s total counts

  sum_x = data.frame(SampleID= colnames(x), sum = colSums(x))

  samples_to_remove =  sum_x$SampleID[sum_x$sum == 0]

  x = x[,!colnames(x) %in%samples_to_remove ]

  #get bacterial counts from metadata
  myenv = data.frame(SampleID = colnames(x))
  myenv$Bacteria_bracken = metadata$Bacteria_bracken[match(myenv$SampleID,metadata$SampleID )]
  myenv$Bacteria_bracken  = as.numeric(myenv$Bacteria_bracken )

  #create first table concerning normalization according to bacterial counts
  myprop = data.frame(Allele_ID = rownames(x))

  for(n in 1:ncol(x)){
    #select column
    myprop.n = x[,n]
    #label column
    mysample.n = myenv$SampleID[which(myenv$SampleID == colnames(x)[n])]
    #take sum of bacterial counts
    mysum = myenv$Bacteria_bracken[myenv$SampleID ==  mysample.n]
    #compute proportion and multiply by 1M
    myprop.n = (myprop.n/mysum)*1000000
    #create data frame
    myprop.n = data.frame(name.x = myprop.n)
    #label column
    colnames(myprop.n) = mysample.n
    #add to the principal table
    myprop = cbind(myprop, myprop.n)

    #remove temporary objects
    rm(myprop.n)
    rm(mysample.n)
    rm(mysum)
    rm(n)

  }

  #normalise by allele length

  myprop_length = as.data.frame(matrix(rep(NA,ncol(myprop)), nrow=1))
  colnames(myprop_length) = colnames(myprop)

  for(i in 1:nrow(myprop)){

    #select allele
    myallele_length= myprop$Allele_ID[i]

    #select values (normalized counts by bacterial reads)
    myprop_length.i = myprop[myprop$Allele_ID == myallele_length,]

    #eliminate first column (non-numeric)
    myprop_length.i = myprop_length.i[,-1]

    #take the length in kb
    myallele_length_kb = mytab_allele_length$length_kb[mytab_allele_length$Allele_ID == myallele_length]

    myprop_length.final.i = myprop_length.i/myallele_length_kb

    myprop_length.final.i$Allele_ID = myallele_length

    #reorder columns
    myprop_length.final.i = myprop_length.final.i[,c(ncol(myprop_length.final.i), 1:(ncol(myprop_length.final.i)-
1))]

    #rbind tables
    myprop_length = rbind(myprop_length,myprop_length.final.i )

    #remove objects
    rm(myprop_length.i)
    rm(myallele_length_kb)
    rm(myprop_length.final.i)
  }

  #remove first row with NAs

  myprop_length =  myprop_length[-1,]

  #add labels for x = table counts
  x$Allele_ID = rownames(x)

  mylist = list(x,myprop, myprop_length, myenv,mytab_allele_length )
  names(mylist) = c("counts_with_no_zero_samples","normalized_without_length", "normalized_by_also_length",
                   "bracken_bacterial_counts", "table_allele_length")

  return(mylist)

}


#computation

only_paired_no_multiple_mapping_norm_list = normalize_data(x = only_paired_no_multiple_mapping, metadata = metada
ta)
```

```
## [1] "Allele_ID and Allele_ID2 contain the same values"
```

```
# "Allele_ID and Allele_ID2 contain the same values"

write.xlsx(only_paired_no_multiple_mapping_norm_list, file = "only_paired_no_multiple_mapping_norm.xlsx")

#save normalized data allele

save(only_paired_no_multiple_mapping_norm_list,file = "allele_only_paired_no_multiple_mapping_norm_list.Rdata")
```

## Function used to collect normalised counts for each ARG gene

The function sums up the normalised counts of all sequences for a given ARGs

```
create_table_gene = function(x){
  #select sheets
  x = x[c("normalized_without_length","normalized_by_also_length" )]

  #create list to collect results
  mylist = list()

  #create a loop for each eelment of the list
  for(h in 1:2){

    y = x[[h]]
    y$Gene = as.character(unlist(sapply(y$Allele_ID, function(x){strsplit(x, split ="_")[[1]][1]})))

    #print number of unique genes
    mygenes = unique(y$Gene )
    print(paste("my unique genes are ", length(mygenes), sep =""))

    #create a table to collect results
    mytable.final = data.frame(matrix(rep(NA, ncol(y)-1), nrow=1))
    colnames(mytable.final) = c("Gene", colnames(y)[which(!colnames(y) %in% c("Allele_ID","Gene"))])

    #sum norm counts for each gene
    for(k in 1:length(mygenes)){
      y.k = y[y$Gene == mygenes[k],]

      #if gene contains one allele
      if(nrow(y.k) == 1){
        mytable.k = y.k[,which(!colnames(y.k) %in% c("Allele_ID","Gene"))]
        mytable.k$Gene =mygenes[k]
        #reorder column
        mytable.k = mytable.k[, c(ncol(mytable.k), 1:(ncol(mytable.k)-1))]

      }else{
        #if gene contains more than one allele
        mytable.k = y.k[,which(!colnames(y.k) %in% c("Allele_ID","Gene"))]
        mytable.k = apply(mytable.k,2, function(z) sum(z))
        mytable.k = as.data.frame(t(as.data.frame(mytable.k)))
        rownames(mytable.k) = NULL
        mytable.k$Gene =mygenes[k]
        #reorder column
        mytable.k = mytable.k[, c(ncol(mytable.k), 1:(ncol(mytable.k)-1))]
      }

      mytable.final=rbind(mytable.final,mytable.k)
    }
    mytable.final= mytable.final[-1,]
    mylist[[h]] = mytable.final
    names(mylist)[h] = names(x)[h]
  }
   return(mylist)
}

gene_only_paired_no_multiple_mapping_norm_list = create_table_gene(x=only_paired_no_multiple_mapping_norm_list)
```

```
## [1] "my unique genes are 2534"
## [1] "my unique genes are 2534"
```

```
library(openxlsx)
write.xlsx(gene_only_paired_no_multiple_mapping_norm_list , file = "gene_only_paired_no_multiple_mapping_norm.xls
x")
save(gene_only_paired_no_multiple_mapping_norm_list,file = "gene_only_paired_no_multiple_mapping_norm.Rdata")
```

## Function used for PCoA plot

```
mypcoa_1var = function(x, variable, metadata){
  require(vegan)
  require(ggplot2)

  #convert metadata variable in character
  metadata[, variable] = as.character( metadata[, variable])

  # built metadata table for PCOA:

  x.env = data.frame(SampleID= colnames(x))
  x.env$SampleID= as.character(x.env$SampleID)
  x.env$variable= metadata[,variable][match(x.env$SampleID, metadata$SampleID)]
  x.env$variable[is.na(x.env$variable)] = "none"
  x.env$variable[x.env$variable == "NA"] = "none"
  x.env$variable[x.env$variable == ""] = "none"
  x.env$variable[x.env$variable == "Missing"] = "none"
  x.env$variable[x.env$variable == "not_known"] = "none"
  x.env$variable[x.env$variable == ".a"] = "none"

  #remove NA values in metadata
  n.samples.before = ncol(x)
  sam_to_remove=  x.env$SampleID[ x.env$variable == "none"]

  #remove samples with no information in variables
  if(identical(sam_to_remove, character(0))){
    x=x
    x.env = x.env

  }else{
    x.env = x.env[!x.env$SampleID %in% sam_to_remove,]
    x = x[, -which(colnames(x) %in% sam_to_remove )]
  }


  #remove species that had 0 counts over the selected samples
  x = x[rowSums(x)>0,]

  #report n of species and no of samples after selection
  n.samples = ncol(x)
  n.species = nrow(x)

  #are datasets aligned?
  if(all(x.env$SampleID == colnames(x))){
    print("metadata and read counts table columns are aligned")
  }else{stop("metadata and read counts table columns are NOT aligned")}

  #transf. sqrt
  y= apply(x, 2, function(x) sqrt(x))

  ## Bray-Curtis distances between samples
  dis <- vegdist(t(y), dist ="bray")

  ## create groups
  groups <- as.factor(x.env$variable)

  ## Calculate multivariate dispersions
  mod <- betadisper(dis, group = groups)
  mydf = as.data.frame(mod$vectors)

  #compute %var axes
  var_values <- round(100*mod$eig / sum(mod$eig), 2)

  mydf$variable = as.character(x.env$variable[match(rownames(mydf),x.env$SampleID)])
  #add centroids
  centroids = as.data.frame(mod$centroids)
  mydf$PCoA1_centr = centroids$PCoA1[match(mydf$variable, rownames(centroids))]
  mydf$PCoA2_centr = centroids$PCoA2[match(mydf$variable, rownames(centroids))]

  mytitle = paste(variable,  n.samples , "out of",  n.samples.before, "on", n.species, "genes", sep =" ")


  pcoa_plot = ggplot(mydf, aes(x = PCoA1, y = PCoA2)) + geom_point(aes(color =variable, alpha = 0.5))+
    ggtitle(mytitle)+
    geom_segment(aes(x=PCoA1_centr, y=PCoA2_centr, xend=PCoA1, yend=PCoA2, alpha = 0.3,color =variable))+
    geom_point(aes(x=PCoA1_centr,y=PCoA2_centr, color =variable),size=5)+
    labs(x = paste("PCoA1 (",round(var_values[1],2), "%)", sep =""),
         y = paste("PCoA2 (",round(var_values[2],2), "%)", sep =""))
  return(pcoa_plot)
}
```

##Show PCoA of resistome according to delivery mode as reported in Figure 1:

```
#select samples
mysamples = metadata$SampleID[metadata$select_correct_resistome == "yes"]
# > length(mysamples)
# [1] 390

resistome_gene_select = gene_only_paired_no_multiple_mapping_norm_list$normalized_by_also_length
rownames(resistome_gene_select) = resistome_gene_select$Gene
resistome_gene_select = resistome_gene_select[,-which(colnames(resistome_gene_select) == "Gene"),]
resistome_gene_select = resistome_gene_select[, mysamples]

# > ncol(resistome_gene_select)
# [1] 390
```

```
## [1] "metadata and read counts table columns are aligned"
```

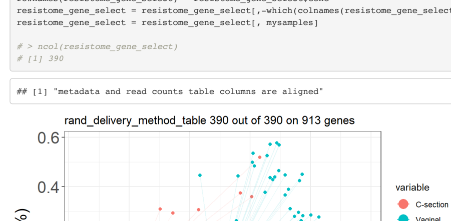


##Function to compute PERMANOVA The function requires to specify which are the continous variables. In our analyses, these were: "bc_pi_gest_w"= gestation in weeks and "ba_weight_bi" = weight at birth

```
run_permanova_global = function(x, metadata, variable,mycontinous_variables){
  require(vegan)

  #PERMANOVA for continuous variables:

  if(variable %in% mycontinous_variables){
    # built metadata

    x.env = data.frame(SampleID= colnames(x))
    x.env$SampleID= as.character(x.env$SampleID)
    x.env$variable= metadata[,variable][match(x.env$SampleID, metadata$SampleID)]

    #remove na values from the x and env
    n.samples.before = ncol(x)

    if(any(is.na(x.env$variable))){

      #which samples contain NA values?

      sample_to_remove = x.env$SampleID[is.na(x.env$variable)]

      x.env = x.env[!x.env$SampleID %in% sample_to_remove,]
      x = x[, colnames(x) %in% x.env$SampleID]

    }else{
      x.env = x.env
      x = x
    }

    #remove species that had 0 counts over the selected samples
    x = x[rowSums(x)>0,]

    #report n of species and no of samples after selection
    n.samples = ncol(x)
    n.species = nrow(x)

    #are datasets aligned?
    if(all(x.env$SampleID == colnames(x))){
      print("metadata and read counts table columns are aligned")
    }else{stop("metadata and read counts table columns are NOT aligned")}

    #variable

    mypermanova = adonis2(sqrt(t(x))~ variable, data = x.env , method ="bray", permutations =9999)

    #collect infos
    mydf = data.frame(n.samples.before = n.samples.before,n.samples.after = n.samples,
                      n.species = n.species,pseudoF =  mypermanova$F[1],  p.value= mypermanova[,"Pr(>F)"][1],
                      r.square = mypermanova$R2[1], by.arg = "terms", variable = variable)

    return(list(mydf, mypermanova))


    #PERMANOVA for categorical variable:
  } else if(!variable %in% mycontinous_variables){

    #convert metadata variable in character
    metadata[, variable] = as.character( metadata[, variable])

    # built metadata

    x.env = data.frame(SampleID= colnames(x))
    x.env$SampleID= as.character(x.env$SampleID)
    x.env$variable= metadata[,variable][match(x.env$SampleID, metadata$SampleID)]
    x.env$variable[is.na(x.env$variable)] = "none"
    x.env$variable[x.env$variable == "NA"] = "none"
    x.env$variable[x.env$variable == ""] = "none"
    x.env$variable[x.env$variable == "Missing"] = "none"
    x.env$variable[x.env$variable == "not_known"] = "none"
    x.env$variable[x.env$variable == ".a"] = "none"

    #remove NA values in metadata
    #report no of samples before the selection
    n.samples.before = ncol(x)
    sam_to_remove=  x.env$SampleID[ x.env$variable == "none"]

    if(identical(sam_to_remove, character(0))){

      x.env = x.env
      x = x

    }else{
      #remove samples with no information for the tested variable
      x.env = x.env[!x.env$SampleID %in% sam_to_remove,]
      x = x[, -which(colnames(x) %in% sam_to_remove )]
    }

    #remove species that had 0 counts over the selected samples
    x = x[rowSums(x)>0,]

    #report n of species and no of samples after selection
    n.samples = ncol(x)
    n.species = nrow(x)

    #are datasets aligned?
    if(all(x.env$SampleID == colnames(x))){
      print("metadata and read counts table columns are aligned")
    }else{stop("metadata and read counts table columns are NOT aligned")}

    #variable

    mypermanova = adonis2(sqrt(t(x))~ variable, data = x.env , method ="bray", permutations =9999)

    #collect infos
    mydf = data.frame(n.samples.before = n.samples.before,n.samples.after = n.samples,
                      n.species = n.species,pseudoF =  mypermanova$F[1],  p.value= mypermanova[,"Pr(>F)"][1],
                      r.square = mypermanova$R2[1], by.arg = "terms", variable = variable)

    return(list(mydf, mypermanova))

  }
}
```

##Function to compute Mantel test:

```
#Prepare data from bacterial composition:

species_bracken_prop_select = species_bracken[, mysamples]
species_bracken_prop_select = apply(species_bracken_prop_select,2, function(x) (x/sum(x)*100))

#Keep species that have a relative abundance higher than 0
keep_species <- rowSums(species_bracken_prop_select) > 0
table(keep_species)
```

```
## keep_species
## FALSE  TRUE
##     5  2803
```

```
# keep_species
# FALSE  TRUE
# 5  2803
species_bracken_prop_select= species_bracken_prop_select[keep_species,]

#Prepare data from normalised counts of ARGs
arg = gene_only_paired_no_multiple_mapping_norm_list$normalized_by_also_length
rownames(arg) = arg$Gene
arg = arg[, -which(colnames(arg) == "Gene")]

#keep samples in common with ARG and MAG

ARG_select <- arg[, colnames(arg) %in% c(intersect(colnames(arg), colnames(species_bracken_prop_select)))]
MAG_select <- species_bracken_prop_select[, colnames(species_bracken_prop_select) %in% c(intersect(colnames(arg),
colnames(species_bracken_prop_select)))]

#Since the analyses is computationally expensive given the high number of samples,the code used is commented and
not run in Rmarkdown

# ARG_select_dist <- vegdist(t(as.matrix(ARG_select)), method = "horn")
# MAG_select_dist <- vegdist(t(as.matrix(MAG_select)), method = "horn")
#
# mytest = mantel(ARG_select_dist, MAG_select_dist, method = "kendall")
# sink("mytest_final_390.txt")
# print(mytest)
# sink()
```

##Function used for DESeq2 analyses

```
run_deseq2 = function(x, metadata, variable){
  require(DESeq2)

  #round values of x

  x = round(x, digits = 0)

  #add 1 to all alleles to avoid zeros
  x = x+1

  #convert metadata variable in character
  metadata[, variable] = as.character( metadata[, variable])

  #built metadata
  x.env = data.frame(SampleID= colnames(x))
  x.env$SampleID= as.character(x.env$SampleID)
  x.env$variable= metadata[,variable][match(x.env$SampleID, metadata$SampleID)]

  x.env$variable[is.na(x.env$variable)] = "none"
  x.env$variable[x.env$variable == "NA"] = "none"
  x.env$variable[x.env$variable == ""] = "none"
  x.env$variable[x.env$variable == "Missing"] = "none"
  x.env$variable[x.env$variable == "not_known"] = "none"
  x.env$variable[x.env$variable == ".a"] = "none"

  colnames(x.env)[colnames(x.env) == variable] = "variable"

  #remove NA values in metadata
  sam_to_remove=  x.env$SampleID[ grep("none",x.env$variable)]
  n.samples.x.before = ncol(x)

  #remove samples with no information in variables
  if(identical(sam_to_remove, character(0))){
    x.env = x.env
    x = x[, colnames(x) %in% x.env$SampleID]


  }else{
    #remove samples with no information for the tested variable
    x.env = x.env[!x.env$SampleID %in% sam_to_remove,]
    x = x[, colnames(x) %in% x.env$SampleID]


  }

  n.samples.x.after = ncol(x)

  if(all(colnames(x)== x.env$SampleID)){
    print("metadata and x are aligned")
    print(paste("no. samples of x: ", n.samples.x.after, " out of ", n.samples.x.before, " selected", sep=""))
  }else{stop("metadata and x are NOT aligned: ERROR")}


  dds <- DESeqDataSetFromMatrix(countData = x,
                                colData = x.env,
                                design= ~ variable)


  dds <- DESeq(dds)
  #resultsNames(dds) # lists the coefficients
  res <- results(dds, name= resultsNames(dds)[grep("Intercept", resultsNames(dds),invert =TRUE)])

  #convert res as data.frame and add taxonomy

  res <- as.data.frame(res)
  res$comparison = rep(resultsNames(dds)[grep("Intercept", resultsNames(dds),invert =TRUE)], nrow(res))

  #add sign for the p-value

  sig = rep("", nrow(res))
  sig[res$pvalue <= 0.05] <-'.'
  sig[res$pvalue <= 0.01] <-'*'
  sig[res$pvalue <= 0.001] <-'**'
  sig[res$pvalue <= 0.0001] <-'***'

  res$sig = sig

  #add sign for the adjusted p-value
  res$ad.sig = rep("", nrow(res))

  ad.sig = rep("", nrow(res))
  ad.sig[res$padj <= 0.05] <-'.'
  ad.sig[res$padj <= 0.01] <-'*'
  ad.sig[res$padj <= 0.001] <-'**'
  ad.sig[res$padj <= 0.0001] <-'***'

  res$ad.sig = ad.sig

  #add number of partecipants the gene is present:

  n.partecipants= c()

  for(g in 1:nrow(res)){
    myallele = rownames(res)[g]
    n.partecipants.g = sum(x[rownames(x) ==  myallele,]>1)
    n.partecipants = c(n.partecipants, n.partecipants.g)
    rm(myallele)
    rm(n.partecipants.g)
  }

  res$n.partecipants = n.partecipants
  res$n.samples.x.before = n.samples.x.before
  res$n.samples.x.after =  n.samples.x.after
  res$gene = rownames(res)
  res$variable = rep(variable, nrow(res))

  #add class

  library(readxl)
  #it is file containing the matching between ARG sequence and resistance class
  #the file was obtained from "phenotype.txt" in ResFinder database
  gene_information = read_xlsx("summary_gene_allele.xlsx")
  gene_information = as.data.frame(gene_information)

  #add class
  res$class = gene_information$class[match(res$gene,gene_information$gene)]

  #re-order columns:

  res = res[, c("variable", "gene", "class", "baseMean",    "log2FoldChange",   "comparison","lfcSE",   "stat", "
pvalue",
                "padj", "sig",  "ad.sig",   "n.partecipants",   "n.samples.x.before",   "n.samples.x.after")]
  return(res)
}

#The following lines of code used to obtain the DESeq2 analyses are commented and not run in Rmarkdown

# variables= c("rand_delivery_method_table","formula_milk","ra_probiotic_yn",
#              "bc_anti_preg_corr", "bc_anti_lab_corr")
#
#
# #create my list
# de_list = list()
#
# for(n in 1:length(variables)){
#
#   de_list[[n]] = run_deseq2(x = resistome_gene_select, metadata = metadata,
#                              variable = variables[n])
#   names(de_list)[n] = variables[n]
# }
#
# library(openxlsx)
# write.xlsx(de_list, file = "deseq2_analyses_resistome.xlsx")
```

##Function used to filter ARGs gene based on log2 fold change and adjusted p-value

```
filter_function = function(x, padj, foldchange){
  #remove NA
  x = x[!is.na(x$padj),]
  x = x[x$padj <  padj,]

  x = x[abs(x$log2FoldChange) >= foldchange,]
  return(x)
}

#The following line of code used to filter data is commented and not run in Rmarkdown

#de_list_select_058 = lapply(de_list, function(x) filter_function(x, padj=0.05, foldchange=0.58))
```

##How obtain the number of differentially abundant genes in a given comparison For simplicity only delivery mode is reported

```
#Code lines are commented and not run in Rmarkdown

#1. how many genes are differentially abundant between the two conditions?
# delivery = de_list$rand_delivery_method_table
#
# table(delivery$padj < 0.05, useNA = "always")
# FALSE  TRUE  <NA>
#   309   119  2106

#2. how many genes are differentially abundant between the two conditions
# and present in at least 100 samples?
#table(delivery$padj < 0.05 & delivery$n.partecipants >= 100)
# FALSE  TRUE
# 2513    21

#3. which are these genes?
#mytopgene_delivery = delivery[delivery$pvalue < 0.05 & delivery$n.partecipants >= 100,]

# library(openxlsx)
# write.xlsx(mytopgene_delivery, file = "mytopgene_delivery_only_paired_no_multiple_mapping_norm_tab_de_list_dese
```

```
q2.xlsx")
```

##Function used for Wilcoxon tests

```
run_wc_test = function(x, metadata, variable, taxonomy, myterms){


  #convert metadata variable in character
  metadata[, variable] = as.character( metadata[, variable])

  #built metadata
  x.env = data.frame(SampleID= colnames(x))
  x.env$SampleID= as.character(x.env$SampleID)
  x.env$variable= metadata[,variable][match(x.env$SampleID, metadata$SampleID)]

  x.env$variable[is.na(x.env$variable)] = "none"
  x.env$variable[x.env$variable == "NA"] = "none"
  x.env$variable[x.env$variable == ""] = "none"
  x.env$variable[x.env$variable == "Missing"] = "none"
  x.env$variable[x.env$variable == "not_known"] = "none"
  x.env$variable[x.env$variable == ".a"] = "none"

  colnames(x.env)[colnames(x.env) == variable] = "variable"

  #remove NA values in metadata
  sam_to_remove=  x.env$SampleID[ grep("none",x.env$variable)]
  n.samples.x.before = ncol(x)

  #remove samples with no information in variables
  if(identical(sam_to_remove, character(0))){
    x.env = x.env
    x = x[, colnames(x) %in% x.env$SampleID]

  }else{
    #remove samples with no information for the tested variable
    x.env = x.env[!x.env$SampleID %in% sam_to_remove,]
    x = x[, colnames(x) %in% x.env$SampleID]

  }

  n.samples.x.after = ncol(x)

  if(all(colnames(x)== x.env$SampleID)){
    print("metadata and x are aligned")
    print(paste("no. samples of x: ", n.samples.x.after, " out of ", n.samples.x.before, " selected", sep=""))
  }else{stop("metadata and x are NOT aligned: ERROR")}

  #before running test, create objects to collect information
  taxa = c()
  name_variable = c()
  w.stats = c()
  p.value = c()
  sig= c()
  n.partecipants= c()
  term1 = c()
  term2 = c()
  median1 = c()
  median2 = c()
  mean1 = c()
  mean2 = c()
  log2FC = c()
  log2FC_def = c()

  #define terms
  myterms= myterms
  samples_term1 = myterms[1]
  samples_term2 = myterms[2]

  for(g in 1:nrow(x)){

    taxa = c(taxa, rownames(x)[g])
    name_variable = c(name_variable, variable)

    #create a second table to link to metadata for WC test

    myrel_abu_taxa = data.frame(relabu = x[g,])
    x.env2 = x.env
    x.env2$taxa = myrel_abu_taxa$relabu[match(x.env2$SampleID, rownames(myrel_abu_taxa ))]

    #compute test
    mytest = wilcox.test(taxa~variable, data =x.env2, paired = FALSE)

    w.stats = c(w.stats,mytest$statistic[[1]] )
    p.value = c( p.value,mytest$p.value[[1]] )


    #median, mean, fc, number part.

    median1.g = median(x.env2$taxa[ x.env2$variable == samples_term1])
    median2.g = median(x.env2$taxa[ x.env2$variable == samples_term2])

    log2FC.g = log(median1.g+0000000000000.1,2)-log(median2.g+0000000000000.1,2)
    log2FC_def.g = paste( samples_term1,  samples_term2, sep ="_vs_"  )

    mean1.g = mean(x.env2$taxa[ x.env2$variable == samples_term1])
    mean2.g = mean(x.env2$taxa[ x.env2$variable == samples_term2])

    n.partecipants.g = nrow(x.env2[x.env2$taxa >0,])

    #bind values

    median1 = c(median1, median1.g)
    median2 = c(median2, median2.g)
    mean1 = c(mean1, mean1.g)
    mean2 = c(mean2, mean2.g)
    log2FC =c(log2FC,log2FC.g)
    log2FC_def = c(log2FC_def,log2FC_def.g)
    n.partecipants = c(n.partecipants,n.partecipants.g)

    rm(median1.g)
    rm(median2.g)
    rm(mean1.g)
    rm(mean2.g)
    rm(log2FC.g)
    rm(n.partecipants.g)
  }

  #add sign for the p-value

  sig = rep("", length(p.value))
  sig[p.value <= 0.05] <-'*'
  sig[p.value <= 0.01] <-'**'
  sig[p.value <= 0.001] <-'***'
  sig[p.value <= 0.0001] <-'****'

  res = data.frame(taxa = taxa,
                   name_variable =name_variable,
                   w.stats = w.stats,
                   p.value = p.value,
                   sig= sig,
                   n.partecipants= n.partecipants,
                   term1 =  rep(samples_term1,length(taxa)),
                   term2 =  rep(samples_term2,length(taxa)),
                   median1 = median1,
                   median2 = median2,
                   mean1 = mean1,
                   mean2 = mean2,
                   log2FC = log2FC,
                   log2FC_def = log2FC_def)

  #add taxonomy merge
  if(!is.null(taxonomy)){
    res$label = taxonomy$phyl_fa_spec[match(res$taxa, taxonomy$merged)]
  }else{
    res$label = as.character(unlist(sapply(res$taxa, function(x) strsplit(x, split ="_&_")[[1]][1])))
  }

  return(res)
}
# The following code lines are commented and not run in the Rmarkdown file

# #build a table with variables and terms
#
# variables= data.frame(variable = c("rand_delivery_method_table","formula_milk","ra_probiotic_yn",
#                                    "bc_anti_preg_corr","bc_anti_lab_corr"))
#
# variables$term1 = c("Vaginal", "yes","Yes", "Yes", "Yes")
# variables$term2 = c("C-section", "no","No", "No", "No")
#
#
# #create list to collect results from species
# de_list_species = list()
#
# for(n in 1:nrow(variables)){
#
#   de_list_species[[n]] = run_wc_test(x = species_prop_select,variable = variables[n,"variable"],
#                                      metadata = metadata, taxonomy = taxonomy_species, myterms = c(variables[n,
"term1"], variables[n,"term2"]))
#
#   names(de_list_species)[n] = variables[n,"variable"]
# }
#
# library(openxlsx)
# write.xlsx(de_list_species, file = "de_list_species.xlsx")
```

##Function used to obtain Figure 2

```
myplot = function(x){
  require(ggplot2)

  #change label variables

  x$variable = factor(x$variable)
  print(levels(x$variable))
  #> levels(x$variable)
  # [1] "bc_anti_lab_corr"           "bc_anti_preg_corr"          "formula_milk"
  # [4] "ra_probiotic_yn"            "rand_delivery_method_table"
  levels(x$variable) = c("IPA","AB_preg",
                         "Formula","Probiotics",
                         "Delivery")

  x$variable = factor(as.character(x$variable), levels = c("Delivery","Formula","Probiotics",
                                                            "AB_preg","IPA"))

  #add class
  x$class = gene_information$class_sl[match(x$gene,gene_information$gene)]
  #label gene with class
  x$label = paste(x$class, x$gene, sep =": ")

  #add stars for the p-values (the signs should be consistent with what reported in other parts of the manuscript
e.g. suppl. fig 5)
  #one star= p <= 0.05; 2 stars = p <= 0.01; 3 stars ***= p <= 0.001; 4 stars ****= p <= 0.0001.

  #add sig_plottingn for the adjusted p-value
  x$ad.sig_plotting = rep("", nrow(x))

  ad.sig_plotting = rep("", nrow(x))
  ad.sig_plotting[x$padj <= 0.05] <-'*'
  ad.sig_plotting[x$padj <= 0.01] <-'**'
  ad.sig_plotting[x$padj <= 0.001] <-'***'
  ad.sig_plotting[x$padj <= 0.0001] <-'****'

  x$ad.sig_plotting = ad.sig_plotting

  x$label_fc = paste(round(x$log2FoldChange,1),x$ad.sig_plotting, sep = " ")

  myggplot =  ggplot(x, aes(x=label, y=variable,color = log2FoldChange, group=class,
                            label= label_fc)) +
    geom_point(aes(size = n.partecipants),
               position=position_dodge(width=0.7))+
    geom_point(aes(size = n.partecipants),shape = 1,colour = "black")+
    #change color fold change
    scale_color_gradient2(midpoint = 0, low = "blue", mid = "white",
                          high = "red", space = "Lab" )+
    geom_text( nudge_x = 0.25, nudge_y = 0.25,
               #hjust=0, vjust=-0.5
               check_overlap = TRUE, colour = "black", size=3)+
    #change label of genes and put axis
    theme(axis.text.x = element_text(angle = 90, vjust = 0.5, hjust=1),
          text = element_text(size = 10))+
    coord_flip()+
    theme_bw()
  mylist = list(myggplot, unique(x$class))
  return(mylist)
}

#de_list_select_058 = myplot(x=de_list_select_058_plotting)
#ggsave("de_list_select_058.pdf",plot = de_list_select_058[[1]], width = 10, height =18)
```

##Function to obtain Supplementary figures of differentially abundant taxa

```
myplot = function(x){
  require(ggplot2)

  #change label variables

  x$variable = factor(x$name_variable)
  print(levels(x$variable))
  # #> levels(x$variable)
  # [1] "bc_anti_lab_corr"           "bc_anti_preg_corr"          "formula_milk"
  # [4] "ra_probiotic_yn"            "rand_delivery_method_table"


  levels(x$variable) = c("IPA", "AB_preg","Formula","Probiotics",
                         "Delivery")

  x$variable = factor(as.character(x$variable), levels = c("Delivery","Formula","Probiotics",
                                                            "AB_preg","IPA"))
  x$log2FC_rounded = round(x$log2FC,2)

  x$ggplot_label = paste(x$log2FC_rounded, x$sig, sep =" ")

  myggplot =  ggplot(x, aes(x=label, y=variable,color = log2FC,
                            label= ggplot_label)) +
    geom_point(aes(size = n.partecipants))+
    #   position=position_dodge(width=0.7))+
    geom_point(aes(size = n.partecipants),shape = 1,colour = "black")+
    #change color fold change
    scale_color_gradient2(midpoint = 0, low = "blue", mid = "white",
                          high = "red", space = "Lab" )+
    geom_text( nudge_x = 0.25, nudge_y = 0.25,
               #hjust=0, vjust=-0.5
               check_overlap = TRUE, colour = "black", size=3)+
    #change label of genes and put axis
    theme(axis.text.x = element_text(angle = 90, vjust = 0.5, hjust=1),
          text = element_text(size = 10))+
    coord_flip()+
    theme_bw()

  return(myggplot)
}

#plotting
# phylum_plotting = myplot(x=de_phylum_plotting)
# family_plotting = myplot(x=de_family_plotting)
# genus_plotting = myplot(x=de_genus_plotting)
# species_plotting = myplot(x=de_species_plotting)
#
#
# ggsave("phylum_plotting.pdf",plot = phylum_plotting, width = 10, height =6)
#
# ggsave("family_plotting.pdf",plot = family_plotting, width = 10, height =6)
#
# ggsave("genus_plotting.pdf",plot = genus_plotting, width = 10, height =6)
#
# ggsave("species_plotting.pdf",plot = species_plotting, width = 10, height =8)
#
```

##Function to obtain Supplementary figures of differentially abundant taxa

```
myplot = function(x){
  require(ggplot2)

  #change label variables

  x$variable = factor(x$name_variable)
  print(levels(x$variable))
  # #> levels(x$variable)
  # [1] "bc_anti_lab_corr"           "bc_anti_preg_corr"          "formula_milk"
  # [4] "ra_probiotic_yn"            "rand_delivery_method_table"


  levels(x$variable) = c("IPA", "AB_preg","Formula","Probiotics",
                         "Delivery")

  x$variable = factor(as.character(x$variable), levels = c("Delivery","Formula","Probiotics",
                                                            "AB_preg","IPA"))
  x$log2FC_rounded = round(x$log2FC,2)

  x$ggplot_label = paste(x$log2FC_rounded, x$sig, sep =" ")

  myggplot =  ggplot(x, aes(x=label, y=variable,color = log2FC,
                            label= ggplot_label)) +
    geom_point(aes(size = n.partecipants))+
    #   position=position_dodge(width=0.7))+
    geom_point(aes(size = n.partecipants),shape = 1,colour = "black")+
    #change color fold change
    scale_color_gradient2(midpoint = 0, low = "blue", mid = "white",
                          high = "red", space = "Lab" )+
    geom_text( nudge_x = 0.25, nudge_y = 0.25,
               #hjust=0, vjust=-0.5
               check_overlap = TRUE, colour = "black", size=3)+
    #change label of genes and put axis
    theme(axis.text.x = element_text(angle = 90, vjust = 0.5, hjust=1),
          text = element_text(size = 10))+
    coord_flip()+
    theme_bw()

  return(myggplot)
}

#plotting
# phylum_plotting = myplot(x=de_phylum_plotting)
# family_plotting = myplot(x=de_family_plotting)
# genus_plotting = myplot(x=de_genus_plotting)
# species_plotting = myplot(x=de_species_plotting)
#
#
# ggsave("phylum_plotting.pdf",plot = phylum_plotting, width = 10, height =6)
#
# ggsave("family_plotting.pdf",plot = family_plotting, width = 10, height =6)
#
# ggsave("genus_plotting.pdf",plot = genus_plotting, width = 10, height =6)
#
# ggsave("species_plotting.pdf",plot = species_plotting, width = 10, height =8)
#
```

##Function used to analyse ecological indexes

```
comp_ind = function(x, mysample, mymetadata, type){
  require(vegan)
  #set seed
  set.seed(201404041)

  if(type == "resistome"){

    y= t(x)

    #compute shannon and richness
    S= apply(y, 1, function(y) sum(y>0))

    shannon = diversity(y, index = "shannon")

    simpson = diversity(y, index = "simpson")

    mytab = cbind(richness = S, shannon = shannon, simpson = simpson)

    rar ="no_rarefaction"
    mylist = list(mytab,rar)

  }else{
    y= t(x)
    raremax <- min(rowSums(y))
    print(paste("minimum number of reads: ",raremax, sep =""))

    rar = rrarefy(y, sample= mysample)

    print(paste("sum of counts in all samples after rarefaction is: ",unique(rowSums(rar)), sep =""))

    #compute richness, shannon and simpson

    S= apply(rar, 1, function(y) sum(y>0))

    shannon = diversity(rar, index = "shannon")

    simpson = diversity(rar, index = "simpson")

    mytab = cbind(richness = S, shannon = shannon, simpson = simpson)

    mylist = list(mytab,rar)
    return(mylist)

  }
}

#The following code lines are not run in Rmarkdown
#Prepare tables
#For ARGs
# resistome_gene = gene_only_paired_no_multiple_mapping_norm_list$normalized_by_also_length
# rownames(resistome_gene) = resistome_gene$Gene
# #remove first column
# resistome_gene = resistome_gene[, colnames(resistome_gene) != "Gene"]
#
# #round counts
# resistome_gene_rounded = apply(resistome_gene, 2, function(x) round(x, digits =0))
#
# #select samples by keeping the 390 neonates selected:
# resistome_gene_rounded_select = resistome_gene_rounded[, colnames(resistome_gene_rounded) %in% mysamples]
# ncol(resistome_gene_rounded_select)
# # [1] 390
# nrow(resistome_gene_rounded_select)
# # [1] 2534

######
#species counts from Kraken/Bracken2

#for counts select samples
# counts_select = species_bracken[,colnames(species_bracken) %in% mysamples]
# ncol(counts_select)
# [1] 390
#
# #Compute ecological indeces for species microbiome
# mygene_diversity = comp_ind(x=resistome_gene_rounded_select,
#                             mysample = NULL,
#                             mymetadata = metadata,
#                             type = "resistome")[[1]]
# colnames(mygene_diversity) = paste("arg",colnames(mygene_diversity), sep ="_")
#
# #Compute ecological indeces for ARGs
# myspecies_diversity = comp_ind(x = counts_select,
#                                mysample = 35000,
#                                mymetadata = metadata,
#                                type = "")[[1]]
# colnames(myspecies_diversity) = paste("species",colnames(myspecies_diversity), sep ="_")

#print the results
#
# metadata_select = metadata[metadata$select_correct_resistome == "yes",]
# nrow(metadata_select)
# # [1] 390
#
# metadata_select = merge(metadata_select, mygene_diversity, by.x="SampleID", by.y = "row.names", all = TRUE, sor
t =FALSE)
# metadata_select = merge(metadata_select, myspecies_diversity, by.x="SampleID", by.y = "row.names", all = TRUE,
sort =FALSE)
# nrow(metadata_select)
# # [1] 390
#
# library(openxlsx)
# write.xlsx(metadata_select, "diversity_ars_species.xlsx")
```

##Function to run wilcoxon tests for ecological indeces Results stored in diversity_ars_species.xlsx, obtained in the previous step, are used to run Wilcoxon tests

```
wilcon_one_variable = function(x, variable, index){

  ##convert metadata variable in character
  x[, variable] = as.character( x[, variable])

  # label and remove NAs in the variable column

  x$variable= x[,variable]
  x$variable[is.na(x$variable)] = "none"
  x$variable[x$variable == "NA"] = "none"
  x$variable[x$variable == ""] = "none"

  x$variable[x$variable == "Missing"] = "none"
  x$variable[x$variable == "not_known"] = "none"
  x$variable[x$variable == ".a"] = "none"

  sam_to_remove=  x$SampleID[ x$variable == "none"]

  if(identical(sam_to_remove, character(0))){
    x = x

  }else{
    #remove samples with no information for the tested variable
    x$SampleID = as.character(x$SampleID)
    x = x[!x$SampleID %in% sam_to_remove, ]
  }

  #make a copy of the index colunm and relabel it

  x$index = x[,index]

  mytest = wilcox.test(index~variable, data = x, paired = FALSE  )

  #add mean, median
  mymedians = t(as.data.frame(tapply(x$index,x$variable, median)))
  rownames( mymedians) = NULL
  mymedians_terms = data.frame(median_term1= colnames(mymedians)[1],median_term2= colnames(mymedians)[2])
  colnames(mymedians) = c("median_term1", "median_term2")

  mymeans = t(as.data.frame(tapply(x$index,x$variable, mean)))
  rownames( mymeans) = NULL
  mymeans_terms = data.frame(mean_term1= colnames(mymeans)[1],mean_term2= colnames(mymeans)[2])
  colnames(mymeans) = c("mean_term1", "mean_term2")


  mydf= data.frame(index = index, variable = variable, n.samples = nrow(x), W = mytest$statistic, p.value = mytes
t$p.value)

  mydf=cbind( mydf,mymedians,mymedians_terms, mymeans,  mymeans_terms )

  return(mydf)

}

#The following code lines are not run in Rmarkdown and are commented.
#
# library(openxlsx)
# #read table counts
# mytab = read.xlsx("diversity_ars_species.xlsx")
#
# myoutcomes = c("rand_delivery_method_table","formula_milk","ra_probiotic_yn",
#                "bc_anti_preg_corr", "bc_anti_lab_corr")
#
# #Collect results for ARGs
# mylist_arg =list()
#
# for(u in 1:length(myoutcomes)){
#   mylist_arg[[u]]= rbind(wilcon_one_variable(x=mytab, variable=myoutcomes[u], index="arg_richness"),
#                          wilcon_one_variable(x=mytab, variable=myoutcomes[u], index="arg_shannon"),
#                          wilcon_one_variable(x=mytab, variable=myoutcomes[u], index="arg_simpson"))
#
#   names(mylist_arg)[u]=myoutcomes[u]
# }
#
# write.xlsx(mylist_arg, file = "WC_test_ecoind_arg.xlsx")
#
#
# #Collect results for species
#
# mylist_species =list()
#
# for(u in 1:length(myoutcomes)){
#   mylist_species[[u]]= rbind(wilcon_one_variable(x=mytab, variable=myoutcomes[u], index="species_richness"),
#                              wilcon_one_variable(x=mytab, variable=myoutcomes[u], index="species_shannon"),
#                              wilcon_one_variable(x=mytab, variable=myoutcomes[u], index="species_simpson"))
#   names(mylist_species)[u]=myoutcomes[u]
# }
#
# write.xlsx(mylist_species, file = "WC_test_ecoind_species.xlsx")
```
